# Grazing weakens competitive interactions between active methanotrophs and nitrifiers modulating greenhouse-gas emissions in grassland soils

**DOI:** 10.1038/s43705-021-00068-2

**Published:** 2021-12-09

**Authors:** Hong Pan, Haojie Feng, Yaowei Liu, Chun-Yu Lai, Yuping Zhuge, Qichun Zhang, Caixian Tang, Hongjie Di, Zhongjun Jia, Cécile Gubry-Rangin, Yong Li, Jianming Xu

**Affiliations:** 1 Institute of Soil and Water Resources and Environmental Science, College of Environmental and Resource Sciences, Zhejiang Provincial Key Laboratory of Agricultural Resources and Environment, Zhejiang University, Hangzhou, 310058 China; 2 National Engineering Laboratory for Efficient Utilization of Soil and Fertilizer Resources, College of Resources and Environment, Shandong Agricultural University, Daizong Road, Tai’an City, Shandong 271018 China; 3 Advanced Water Management Centre, The University of Queensland, St Lucia, QLD 4072 Australia; 4 Department of Animal, Plant and Soil Sciences, Centre for AgriBioscience, La Trobe University, Bundoora, VIC 3086 Australia; 5 State Key Laboratory of Soil and Sustainable Agriculture, Institute of Soil Science, Chinese Academy of Sciences, Nanjing, 210008 China; 6 School of Biological Sciences, University of Aberdeen, Aberdeen, AB24 3UU UK

**Keywords:** Soil microbiology, Biogeochemistry

## Abstract

Grassland soils serve as a biological sink and source of the potent greenhouse gases (GHG) methane (CH_4_) and nitrous oxide (N_2_O). The underlying mechanisms responsible for those GHG emissions, specifically, the relationships between methane- and ammonia-oxidizing microorganisms in grazed grassland soils are still poorly understood. Here, we characterized the effects of grazing on in situ GHG emissions and elucidated the putative relations between the active microbes involving in methane oxidation and nitrification activity in grassland soils. Grazing significantly decreases CH_4_ uptake while it increases N_2_O emissions basing on 14-month in situ measurement. DNA-based stable isotope probing (SIP) incubation experiment shows that grazing decreases both methane oxidation and nitrification processes and decreases the diversity of active methanotrophs and nitrifiers, and subsequently weakens the putative competition between active methanotrophs and nitrifiers in grassland soils. These results constitute a major advance in our understanding of putative relationships between methane- and ammonia-oxidizing microorganisms and subsequent effects on nitrification and methane oxidation, which contribute to a better prediction and modeling of future balance of GHG emissions and active microbial communities in grazed grassland ecosystems.

## Introduction

Methane (CH_4_), as the second most potent greenhouse gas (GHG) after carbon dioxide (CO_2_), is considered to be responsible for ~20% of the anthropogenic global warming effect [1]. Its atmospheric concentration has doubled from about 700 ppb at pre-industrial times to the current concentration of 1850.5 ppb [2]. Aerobic oxidation of CH_4_ in soils by methane‐oxidizing bacteria (MOB), also known as methanotrophs, represents the largest biological sink for atmospheric CH_4_ [3]. Methanotrophs have the unique ability to grow on CH_4_ as their sole source of carbon and energy. They are ubiquitous in the environment and play a major role in the removal of the greenhouse gas methane from the biosphere before it is released into the atmosphere [4].

The key step of aerobic methane oxidation, the initial oxidation of CH_4_ to methanol, is catalyzed by the methane monooxygenase which exists either as a particulate, membrane-bound form (pMMO), or a soluble, cytosolic form [5]. It is noticeable that pMMO of MOB and ammonia monooxygenase (AMO) of ammonia-oxidizing bacteria (AOB) are homologous members [6], which are grouped into the copper-containing membrane-bound monooxygenase (CuMMO) family [7]. The AMO of ammonia-oxidizing archaea (AOA) is also a CuMMO, but it is a phylogenetically distant one from those of MOB and AOB [8]. Traditionally, ammonia (NH_3_) was converted into nitrite by AOA/AOB and further oxidized into nitrate by nitrite-oxidizing bacteria (NOB). The evolutionary links between MOB and AOB, together with the similar molecular structure of their substrates (NH_3_ and CH_4_, respectively), lead to functional similarities enabling them to oxidize both NH_3_ and CH_4_, although neither AOB nor MOB are capable of growing on the alternative substrate [9–11].

Methanotrophs and ammonia oxidizers are considered as strong competitors for N to maintain their growth and activity [12, 13]. However, response and activity of methanotrophs to N levels remains unsolved and is still debated. Previous studies either demonstrated that ammonium could stimulate [12, 14–17], suppress [18, 19], or exert little impact on [20, 21] the global methanotrophic CH_4_ sink. Therefore, there are some gaps of knowledge concerning the mechanisms controlling the relationships between methane oxidation and N levels. Additionally, N levels are particularly important in controlling niche specialization of the ecology and evolution of AOA and AOB [14, 22]. AOA generally dominates ammonia oxidation in N-limited soils while AOB dominates ammonia oxidation in N-rich environments [12, 14, 22]. It is thus hypothesized that ammonium-assimilating MOB decreases nitrification rate by competing for N with AOA and AOB, especially in N-limited soils.

Grasslands are known as important biological sinks of CH_4_ and sources of N_2_O and grassland soils are often viewed as important ecosystems influencing global environmental change through their strong capacity to produce, store, and cycle C and N substrates [23, 24]. It is reported that grassland ecosystems contain more than one-third of above- and below-ground C reserves because grasslands cover nearly half of the Earth’s land surface [25]. Notably, management practices of grasslands can alter the exchange of C and N between atmosphere and soil, aboveground and belowground biomass. Livestock grazing, for example, is the most important and pervasive practice in grasslands. Increasing stocking rates have been implemented to meet the increasing food demands of a growing population, but grazing modifies C and N cycling in grassland soils [26]. Grazing has also been shown to be an important factor regulating the emission and uptake of greenhouse gases [27] with moderately-grazed grassland having high CH_4_ uptake, while heavy grazing suppresses soil CH_4_ uptake and stimulates N_2_O emissions [28, 29]. Analysis of the active methanotrophs in such ecosystems has shown that grazing decreases the abundance and diversity of the active methanotrophs in steppe soils [30]. Grazing also significantly decreases nitrification activity, and different grazing intensity leads to diverse active communities with AOA and AOB dominating nitrification in lightly- and heavily-grazed soils, respectively [31]. However, previous studies have primarily focused on effects of grazing on nitrification or methane oxidation, but very little is known about their relationships in grassland ecosystem as well as the responsible microbial communities.

In this study, we firstly investigated in situ greenhouse gas emissions in ungrazed and grazed grasslands for 14-months (August 2014 to October 2015) using closed static chambers. Then a controlled incubation experiment was conducted in the laboratory to understand microbial relationships modulating greenhouse gas emissions in grassland by performing DNA-SIP and Illumina HiSeq 16S ribosomal RNA (rRNA) gene sequencing. The present study aimed to (1) characterize the impact of long-term grazing on active methanotrophs and nitrifiers, (2) examine the mechanisms underlying the effects of N level on CH_4_ and NH_3_ oxidation, and (3) clarify the relationships occurring between important players of the methane and ammonia oxidation in grazed grassland soils. We hypothesized that grazing would suppress the competitions between methane- and ammonia-oxidizing microorganisms by decreasing their diversities and activities. Elucidating these putative relationships is crucial for understanding the methane-nitrogen cycling and its effects on global climate change.

## Materials and methods

### Site description and soil sampling

The experimental site was situated at the Inner Mongolia Grassland Ecosystem Research Station (IMGERS, 43°37′N, 116°43′ E) on the Xilingol steppe of the Xilin River basin, bounded by the west side of the Daxing-An Mountain. This ecoregion possesses a semi-arid continental climate with mean annual temperature of −0.4 °C, and 348 mm of precipitation which was distributed unevenly across the seasons, falling mostly during June–August. The vegetation type was *Leymus chinensis* with some *Stipa grandis* and *Cleistogenes squarrosa*. The soil was classified as Calcic Chernozem according to ISSS Working Group RB, 1998.

Two adjacent long-term field plots were established at the Research Station, one ungrazed and enclosed since 1983 (“Ungrazed”) and the other subjected to free grazing (“Grazed”). Both sites were derived from a paddock that had uniform fertility and slope. At each site, a transect of 60 m × 400 m was established with five equal-sized replicate plots of dimension 60 m × 70 m. A 10-m-wide buffer strip was established between every plot to avoid interactions and allow for sampling. Soil samples were collected from the upper 10-cm layer from five random locations within each plot following an “S” sampling pattern using a 5-cm diameter soil auger in August 2016. The five samples from each plot were grouped into a single composite sample, packed with ice packs, and transported to the laboratory. Soil samples were then passed through a 2-mm sieve. Subsamples were air-dried for physicochemical analysis. The remaining fresh soils were used for the incubation experiment.

Soil pH was measured using a pH meter (Mettler-Toledo, Switzerland) after shaking the soil at a soil-water ratio of 1:2.5. Gravimetric soil moisture content was analyzed by oven-drying at 105 ± 2 °C for 24 h. Soil bulk density was measured by the volumetric ring method according to Lampurlanés and Cantero-Martinez [32]. Soil organic matter was determined by dichromate digestion [33]. Total C and N were determined by dry combustion in a Vario Max CNS analyzer (Elementar Instrument, Mt. Laurel, NJ). Olsen P was extracted by 0.5 M NaHCO_3_ (pH = 8.5) for 0.5 h and determined with the molybdenum blue method [34]. Available potassium was extracted by 1 M ammonium acetate and determined by flame emission spectrophotometry. Exchangeable NH_4_^+^ and NO_3_^−^ were extracted with 1 M KCl for 1 h and determined by a flow injection analyzer (SAN++, Skalar, Holland).

### In situ CH_4_ and N_2_O measurements

To elucidate the effects of grazing on N_2_O and CH_4_ emissions from grassland soils, we collected gas samples using closed-chamber greenhouse gas-flux method from August 2014 to October 2015. For gas sampling, a static chamber (50 cm diameter) was installed in each of the five replicated plots into the ungrazed and grazed soils (in close proximity to the soil sampling points) resulting in ten chambers. Gas samples (40 ml) were collected five times during the in-situ field study, in May (early pasture-growing season, 2015 only), August (peak pasture-growing season), and October (non-growing season) in both 2014 and 2015. Gas samples were collected 0 and 40 min after chamber closure using a 20-ml syringe and injected into preevacuated 20-ml glass bottles and samples were collected between 10 and 12 a.m. The concentrations of N_2_O and CH_4_ in gas samples were analyzed using a gas chromatograph (Shimadzu GC-2010 Plus, Japan).

### Construction and sampling of soil microcosms

Fresh soil (equivalent to 6.0 g dry weight) was pre-incubated at 40% field moisture capacity in a 120-ml serum bottle for ten days at 25 °C in darkness before the incubation experiment. Microcosms were established using seven treatments (each in triplicate, see details in Table S1 including (i) ^13^C-CH_4_ without urea addition (U0 + ^13^CH_4_); (ii) Low ^13^C-urea with ^13^C-CO_2_ (U20 + ^13^CO_2_, corresponding to 20 mg ^13^C-urea-N  kg^−1^ soil); (iii) High ^13^C-urea with ^13^C-CO_2_ (U100 + ^13^CO_2_, corresponding to 100 mg ^13^C-urea-N kg^−1^ soil); (iv) Low ^13^C-urea with ^13^C-CO_2_ and ^13^C–CH_4_ (U20 + ^13^CO_2_ + ^13^CH_4_); (v) High ^13^C–urea with ^13^C-CO_2_ and ^13^C–CH_4_ (U100 + ^13^CO_2_ + ^13^CH_4_); (vi) Low ^12^C–urea with ^12^C-CO_2_ and ^12^C–CH_4_ (U20 + ^12^CO_2_ + ^12^CH_4_); (vii) High ^12^C–urea with ^12^C-CO_2_ and ^12^C–CH_4_ (U100 + ^12^CO_2_ + ^12^CH_4_). The microcosms supplemented with ^13^C–CH_4_ or ^13^C–urea microcosms were used to monitor the active methanotrophs and nitrifiers, respectively, while the microcosms supplemented with ^13^C–urea and ^13^C-CH_4_ were used to monitor the putative relationships between active methanotrophs and nitrifiers. The application rate of U20 was to mimic the annual total N deposition in the grassland ecosystems. The annual total inorganic N deposition to Inner Mongolia grassland regions ranged from 4 to 20 kg N ha^−1^ year^−1^ [35], which is equivalent to 3–15 mg N kg^−1^ dry soil, assuming an effective soil depth of 15 cm. The treatment of U100 was implemented to simulate N input accompanied with livestock excrements, as up to 300 kg N ha^−1^ y^−1^ can be returned to grassland soils in the form of livestock urine [36]. Urea was added weekly by dropwise addition of freshly made urea solution, as degradation of urea into NH_4_^+^-N would occur very quickly in soils, to achieve 60% field capacity to establish a substrate-rich environment for nitrifying communities. For the U0 + ^13^CH_4_ treatment, distilled water was added instead of urea solution to achieve the 60% field capacity. Microcosms were flushed before any supplementation of urea or methane with synthetic air (20% O_2_, 80% N_2_) for 1 min to maintain oxic conditions. The bottles were then sealed with rubber stoppers and aluminum caps and 5% and 1% (v/v) of labeled or unlabeled CO_2_ and CH_4_ were injected into each corresponding microcosm through the rubber septum. The unlabeled and labeled urea and CO_2_ (99 atom% ^13^C) were purchased from the Shanghai Engineering Research Center of Stable Isotopes (Shanghai, China). The unlabeled and labeled CH_4_ (99 atom% ^13^C) were purchased from Sigma-Aldrich (St Louis, MO, USA). The microcosms were incubated at 25 °C in the dark for 21 days. For the ^13^CH_4_ microcosms, the concentrations of CH_4_ were measured daily by gas chromatography (Shimadzu GC-2010 Plus, Japan), and ^13^CH_4_ or ^12^CH_4_ was then renewed to maintain 1% CH_4_ in the microcosms. The measurement time and dynamic consumption of CH_4_ are detailed in Table S2 and Fig. S1. The methane oxidation activity was assessed by measuring the amount of methane oxidized over the 21-day incubation time. Initial soil samples (day 0) were collected immediately after the 10-day pre-incubation and urea addition. Destructive sampling was carried out at 7, 14, and 21 days. About 2 g of soil was sampled from each triplicate microcosm and transferred immediately to a −80 °C freezer for later molecular analysis. Soil NO_3_^−^ and NH_4_^+^ were extracted with 1 M KCl and determined by a flow injection analyzer (SAN++, Skalar, Holland). The net nitrification activity was assessed by the net increase in nitrate concentration over 21 days of incubation.

### Nucleic acid extraction and SIP fractionation

DNA was extracted from 0.5 g of fresh soil with a FastDNA Spin Kit for Soil (MP Biomedicals, LLC., Solon, OH, USA), in accordance with the manufacturer’s protocol. The DNA size and integrity were checked by electrophoresis on a 0.7% agarose gel, and the quantity and purity were estimated using a Nanodrop^®^ND-2000 UV–Vis Spectrophotometer (NanoDrop Technologies, Wilmington, DE, USA). The extracted DNA was stored at −20 °C until further analysis.

Extracted DNA from ^12^C-, ^13^C-CO_2_ and ^12^C-, ^13^C-CH_4_ incubations was subjected to isopycnic density gradient centrifugation to separate the ^13^C-labeled DNA from the ^12^C-labeled DNA in triplicate SIP microcosms. In brief, ~3.0 µg of the DNA extract was mixed with CsCl stock solution to achieve an initial CsCl buoyant density of 1.725 g ml^−1^, by adding a small amount of gradient buffer (GB) or CsCl solution. The isopycnic density gradient centrifugation was performed using a 5.1-ml Quick-Seal polyallomer ultracentrifugation tube in a Vti65.2 vertical rotor (himac CP80NX, Hitachi Koki Co., Ltd. Japan) at 177,000 *g* for 44 h at 20 °C. CsCl density gradients displaced with sterile water from the top of the ultracentrifuge tube were fractionated into equal volumes using an NE-1000 single syringe pump (New Era Pump Systems Inc., Farmingdale, NY, USA) with a precisely controlled flow rate of 0.38 ml min^−1^. A total of 15 DNA gradient fractions were obtained with about 380 µl in each fraction, and 65 µl of each fraction was used for measurement of the refractive index using an AR200 digital hand-held refractometer (Reichert Inc., Buffalo, NY, USA). The fractionated DNA was purified with PEG precipitation (polyethylene glycol 6000) and 70% ethanol, and dissolved in 30 µl of sterile water.

### Quantitative PCR (qPCR) of the *amoA* and *pmoA* genes

The qPCR assays targeting bacterial and archaeal *amoA* genes, as well as *pmoA* genes of total DNA extracts and DNA gradient fractions, were carried out in triplicate with LightCycler 480 (Roche Applied Science). The primers and PCR conditions used are detailed in Table S3. Each qPCR was performed in a 20-µl reaction mixture containing 10 µl SYBR Premix Ex Taq (TaKaRa, Dalian, China), 0.5 µM of each primer and 1 µl of DNA template (1–10 ng) and an appropriate amount of milli-Q water to make a total volume of 20 µl. Melting curve analysis was performed at the end of each real-time PCR run to confirm PCR product specificity, by measuring fluorescence continuously with the temperature increasing from 50 °C to 99 °C. High efficiencies ranging between 80.5 and 108.3% were obtained for amplification of the functional genes, with *R*^2^ values ranging between 0.990 and 0.998.

### Illumina HiSeq sequencing and phylogenetic analysis

High-throughput sequencing of the V4 region of the 16S rRNA gene (amplified using the universal 515F-907R primer set) was used to assess the microbial community composition, including methane-oxidizers and nitrifiers. The HiSeq sequencing was carried out using the total DNA extracted from soil microcosms on day 0 and day 21 and the fractions 3–12 of the labeled (^13^CO_2_ and ^13^CH_4_) and control (^12^CO_2_ and ^12^CH_4_) microcosms at Day 21 (Tables S4, S5). Purified amplicons were pooled in equimolar and paired-end sequenced using an Illumina HiSeq 2500 (PE250) platform. The raw fastq files were demultiplexed, quality-filtered by Trimomatic and merged by FLASH [37] according to the following criteria: (i) the reads were truncated at any site receiving an average quality score <20 over a 50-bp sliding window; (ii) sequences whose overlap was longer than 10 bp were merged according to their overlap sequence; (iii) sequences of each sample were separated according to barcodes (exactly matching) and primers (allowing two nucleotide mismatching), and reads containing ambiguous bases were removed. UPARSE was used to cluster cleaned sequences into operational taxonomic units (OTUs) at 97% similarity threshold. One representative sequence of each OTU was selected to determine taxonomic identification by RDP Classifier algorithm against the Silva (SSU128) 16S rRNA database. The 16S rRNA genes affiliated with aerobic MOB were selected based on two phyla: *Proteobacteria* and *Verrucomicrobia*. Of which, aerobic *Proteobacterial* MOB was divided into two major groups mainly based on phylogeny type I (*Gammaproteobacteria*) and type II (*Alphaproteobacteria*). Type I MOB harboring the family *Methylococcaceae*, which were further divided into type Ia (including genera *Methylosarcina*, *Methylobacter*, *Methylomonas*, *Methylomicrobium*, *Methylosoma*, *Methylosphaera* and *Methylovulum*) and type Ib (including genera *Methylococcus*, *Methylocaldum*, *Methylogaea*, *Methylohalobius,* and *Methylothermus*). Type II MOB include the family *Methylocystaceae* (including genera *Methylocystis* and *Methylosinus*) and *Beijerinckiaceaea* (including genera *Methylocella*, *Methylocapsa*, and *Methyloferula*). MmoX, a subunit of soluble methane monooxygenase, was not accounted for in the 16S sequence analysis. One phylum (*Thaumarchaeota)* was suggested for AOA. Comparative 16S rRNA sequence analyses of cultured AOB revealed that members of this physiological group are confined to two monophyletic lineages within *β‐* and *γ‐proteobacteria*. The former lineage includes the genera *Nitrosomonas* and *Nitrosospira* and the latter the genera *Nitrosococcus*, *Nitrosacidococcus* and *Nitrosoglobus*. All reads classified as belonging to the genera *Nitrococcus*, *Nitrospina*, *Nitrobacter,* and *Nitrospira* were used for NOB phylogenetic analysis.

A representative sequence was used from each OTU of the 16S rRNA for phylogenetic analysis. Phylogenetic analysis of archaeal and bacterial *amoA* genes, NOB, as well as pmoA genes of total DNA and of 13C-labeled DNA, was then conducted by Molecular Evolutionary Genetic Analysis software (MEGA6.0) with 1000-fold bootstrap support. The tree topology was checked by the neighbor-joining algorithm and the minimum evolution method [38]. The entire dataset of 16S rRNA gene reads of total and fractionated DNA was deposited in NCBI’s Sequence Read Archive under BioProject accession numbers PRJNA594293 and PRJNA594236, respectively.

Since it is insufficient to reveal the changes of actual taxon abundances based only on those of relative abundances by high-throughput sequencing analysis [39, 40], the estimated absolute abundance (EAA) of each microbial genus considering both the corresponding relative abundance and the absolute corresponding cell numbers was essential for the comprehensive analysis of the microbial community [39–41]. The EAA was calculated by multiplying the relative abundance (estimated by the sequencing) by the estimated absolute corresponding cell numbers (estimated by qPCR) [41].

### Statistical analysis

One-way analysis of variance followed by Duncan’s multiple range test was used to check for the significant differences between treatments for abundance of functional genes, nitrification potential, and methane-oxidizing potential using SPSS software (version 20). The independent samples *t* test was performed with SPSS 20 (IBM) to check for the significant differences between grazed and ungrazed soils for soil physicochemical properties and *α*-diversity indices. *P* < 0.05 was considered to be statistically significant. The *α*-diversity indices of active methane-oxidizing bacteria and nitrifying communities were calculated using the “vegan” packages in the R environment. Network analysis was performed by CoNet of cytoscape 3.6.1 based on Spearman’s correlation for labeled communities of methane-oxidizers and nitrifiers in microcosms incubated at low and high levels of urea with or without methane supplementation in ungrazed and grazed soils. A co-occurrence event was validated when the Spearman’s coefficient was greater than 0.65 and the *P* value was lower than 0.01. Nodes in the microbiome network represented individual microbial taxa (OTUs), and edges corresponded to the pairwise correlations between nodes. The network visualization was generated by Gephi 0.9.2 [42].

## Results

### Greenhouse-gas emissions, methane, and ammonia oxidation activity

**Fig. 1:**
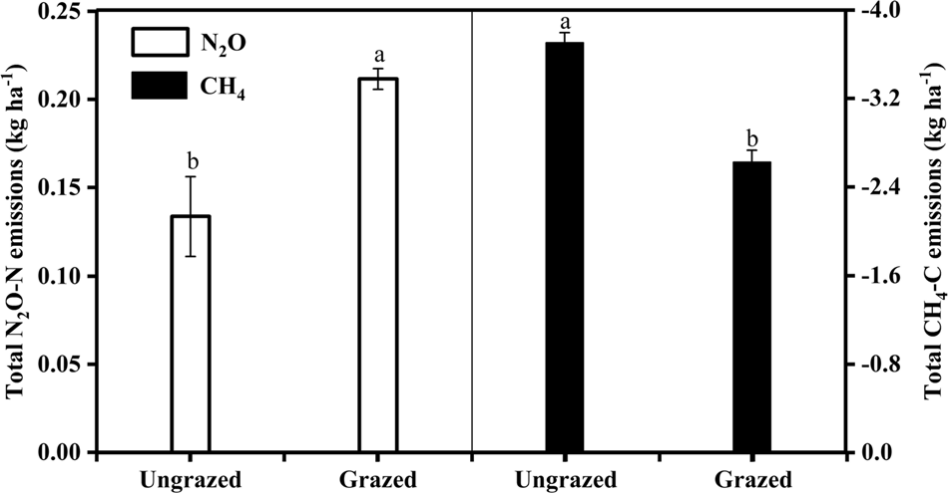
Total N_2_O and CH_4_ emissions from August 2014 to October 2015 in ungrazed and grazed soils. The vertical bars indicate the standard error of the mean (S.E.M.).

The in situ GHGs measurements revealed that total N_2_O emission was 0.134 kg ha^−1^ in the ungrazed soils over the two years, and was significantly lower than that in the grazed soils (0.212 kg ha^−1^) (*P* < 0.01) (Fig. 1). Total CH_4_ emission was −3.7 kg ha^−1^ in the ungrazed soils, and was significantly lower than that in grazed soils (−2.6 kg ha^−1^).

**Fig. 2:**
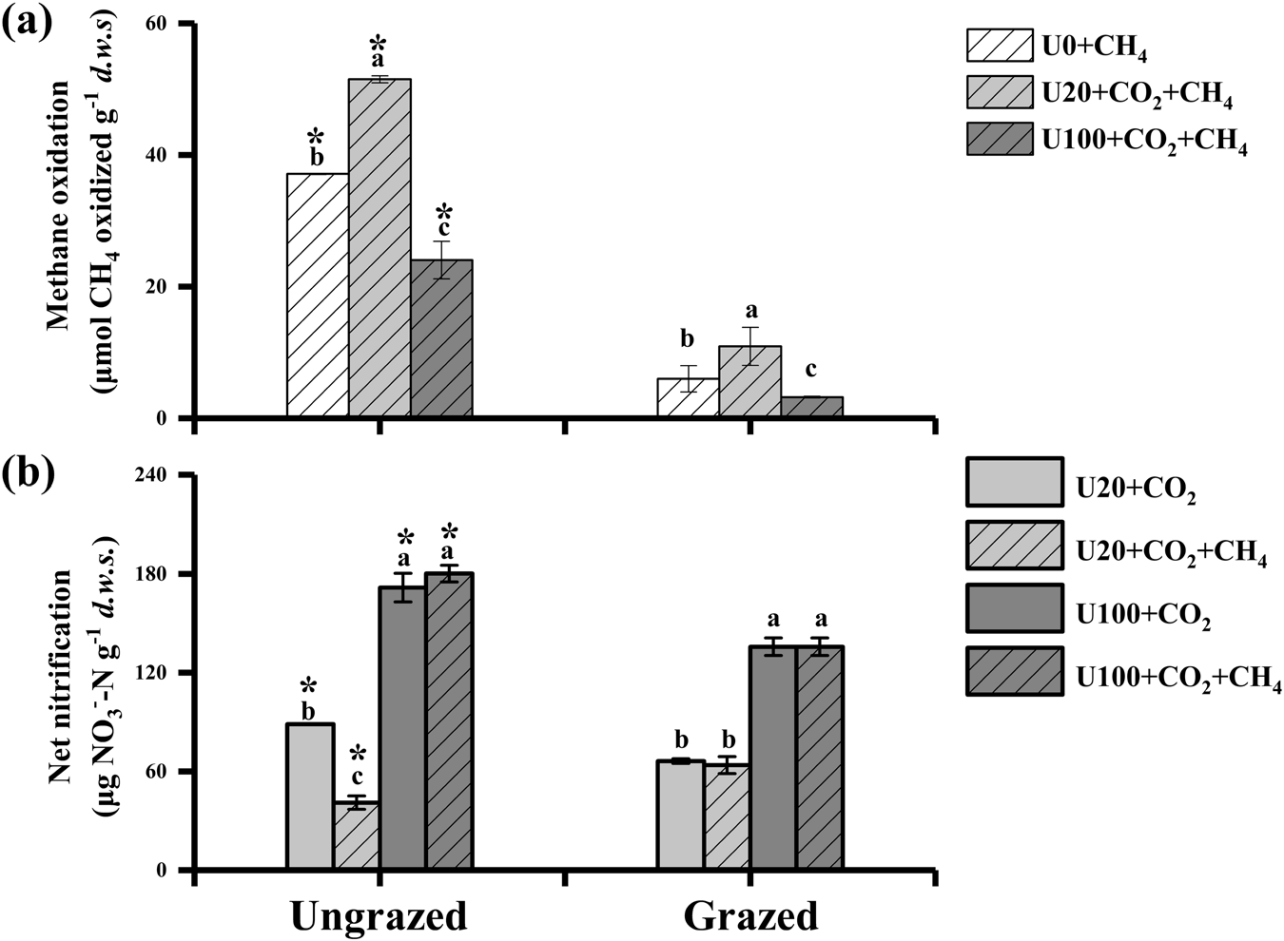
Interactions between methane and ammonia oxidation in grassland soils. Methane oxidation (**a**) and nitrification (**b**) after incubation for 21 days in the ungrazed and grazed soils at weekly urea addition of 0 (U0), 20 (U20), and 100 μg N g^-1^ (U100) in presence or absence of methane addition (1% CH_4_). Methane oxidation was calculated by methane consumption in the microcosms in presence or absence of urea addition, while nitrification was assessed by nitrate production. The error bars represent standard errors of three replicates for the U0 + CH_4_ treatments, and six replicates for the U20 and U100 treatments (^12^C + ^13^C-labeled treatments). Different lower-case letters indicate significant differences among different treatments. Asterisks indicate significant difference between ungrazing and grazing under the same treatment. *d.w.s*. refers to dry weight of soil.

In the incubated soils for 21 days in presence of CH_4_, a total of 37, 51, and 24 µmol CH_4_ g^−1^ dry soil was oxidized in ungrazed soils, while 6, 11, and 3 µmol CH_4_ g^−1^ dry soil was oxidized in grazed soils under supplementation of 0, 20, and 100 µg N g^−1^ of urea (Fig. 2a). Accordingly, the methane oxidation was significantly stimulated in the U20 + CH_4_ treatments but significantly suppressed in the U100 + CH_4_ treatments compared to U0 + CH_4_ soils during the incubation period for both ungrazed and grazed soils. The inconsistency in methane addition resulted from different methane oxidation abilities in these treatments, as we keep 1% CH_4_ in the headspace of the microcosms. The inconsistency in methane addition just indicated different functional activity of methanotrophs in these treatments. The less amount of methane addition in U100-treated soils compared with that in U0 and U20-treated soils was ascribed to the fact that high N levels (U100) significantly depressed methane oxidation activity.

In both ungrazed and grazed incubated soils, net nitrification rate was higher in soil incubated with higher concentrations of urea (Fig. 2b), with a maximum of 180 and 136 µg NO_3_^−^ g^−1^ dry soil in U100-supplemented soils in ungrazed and grazed soils, respectively. The nitrate concentration in the soils increased during the incubation period regardless of treatments, and ammonium decreased conversely over time with a sharper decrease during the first 7-days of the incubation (Fig. S2). Methane supplementation did not impact nitrification rate, apart in U20-supplemented ungrazed soils, for which nitrification was reduced from 89 to 41 µg g^−1^ following methane addition (Fig. 2b).

### Abundance of methylotrophic and nitrifying communities

**Fig. 3:**
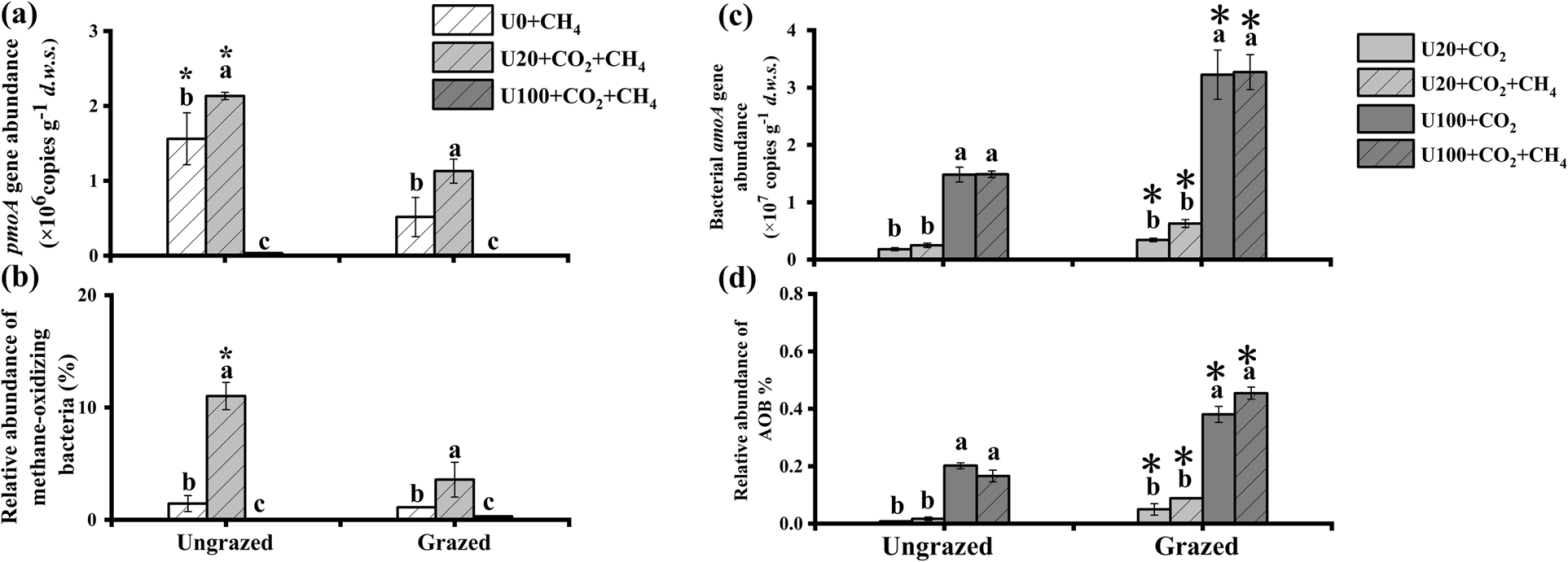
Changes of abundance of methylotrophs and AOB *amoA* genes in soil microcosms over a 21-days incubation in ungrazed and grazed soils following weekly urea addition of 0 (U0), 20 (U20), and 100 μg N g^−1^ (U100) in presence or absence of methane addition (1% CH_4_). The *pmoA* gene abundance (**a**), the relative abundance of targeted 16S rRNA genes affiliating with methane-oxidizing bacteria (**b**), the *amoA* gene copy numbers in total DNA (**c**), relative frequencies of targeted 16S rRNA genes affiliating with ammonia-oxidizing bacteria in total reads (%) (**d**) in the ungrazed and grazed soils after 21-day incubation were represented. The error bars represent standard errors of three replicates for the U0 + CH_4_ treatments, and 6 replicates for the U20 and U100 treatments (^12^C + ^13^C-labeled treatments) (**a**–**c**). The error bars represent standard errors of six replicates for the U20 and U100 treatments (^12^C + ^13^C-labeled treatments) (**d, e**). Different lower-case letters indicate significant differences among different treatments. Asterisks indicate significant difference between ungrazing and grazing under the same urea treatment. *d.w.s*. refers to dry weight of soil.

The abundance of *pmoA*-related organisms increased during the incubation under low urea supplementation (U20) in both ungrazed and grazed soils, but decreased dramatically for the high urea supplementation treatment (U100) compared to the control treatment (Fig. 3a; Table S6). A similar trend was observed for the relative abundance of methylotrophs estimated by 16S rRNA gene sequencing in microcosms incubated for 21 days (Fig. 3b; Table S6). The relative abundance of 16S rRNA genes affiliated with methane-oxidizing bacteria in both ungrazed and grazed soils was significantly stimulated by the low rate of urea addition (20 µg N g^−1^), and decreased following the addition of 100 µg urea-N g^−1^ compared to those without urea addition.

The abundance of bacterial *amoA* gene increased significantly over time in both ungrazed and grazed soils supplemented with high urea (Fig. 3c; Table S7). After incubation, the bacterial *amoA* gene abundance was significantly higher in U100- than in U20-treated soils. Methane addition did not affect the bacterial *amoA* gene abundance. The dynamic changes of the relative abundance of 16S rRNA genes affiliated with AOB were similar to the variations of bacterial *amoA* genes copy numbers under urea and methane treatments in both ungrazed and grazed soils (Fig. 3e; Table S7). By comparison, irrespective of methane addition, the abundance as well as the relative abundance of *Thaumarchaeota* (including all known AOA) decreased significantly during the incubation period with urea addition in both ungrazed and grazed soils (Fig. S3).

In ungrazed soils, the relative abundance of targeted 16S rRNA genes affiliated with NOB increased over time in U20, U100, and U100 + CH_4_ microcosms, but remained constant in U20 + CH_4_ microcosms (Fig. S4a). On the contrary, in grazed soils, the relative abundance of NOB only increased in U100-treated microcosms (Fig. S4b).

### Active methane-oxidizing bacteria and nitrifying communities

**Fig. 4:**
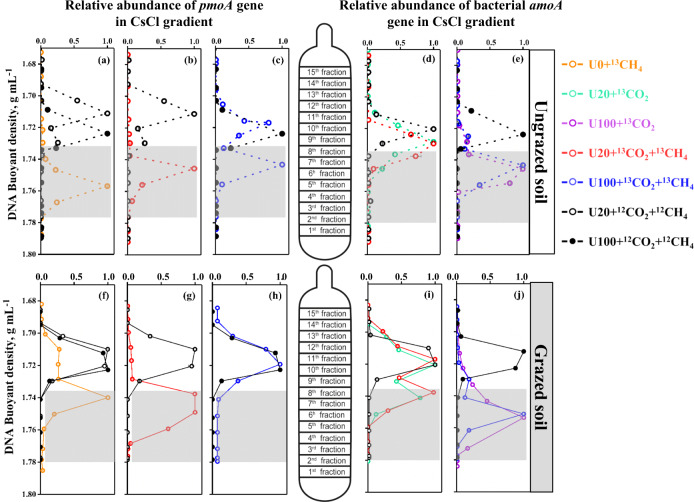
Distribution of* pmoA* and *amoA* gene copy numbers in fractionated DNA across the buoyant density. Quantitative distribution of *pmoA* genes (**a**-**c**, **f**-**h**), and bacterial *amoA* genes (**d**, **e**, **i**, **j**) across the entire buoyant density gradient of the fractionated DNA from the ungrazed (**a**-**e**) and grazed (**f**–**j**) soils at weekly urea addition of 0 (U0), 20 (U20), and 100 μg N g^-1^ (U100), in presence or absence of methane addition (1% CH_4_) after incubation for 21 days. The normalized data are shown as the ratio of the gene copy number in each fraction to the maximum quantities in each treatment. The dotted and plain lines represent samples in ungrazed and grazed soils, respectively.

Isopycnic gradient centrifugation was conducted on the total DNA from each treatment to detect which putative autotrophic methane- and ammonia- oxidizers were incorporating ^13^C-CH_4_ or ^13^C-CO_2_ during the 21-day incubation (Fig. 4). The buoyant density in the 15 fractions ranged from 1.676 to 1.786 g ml^−1^ from the top to the bottom of the ultracentrifugation tube. The abundances of *pmoA* and *amoA* genes in the 15 fractions were determined by specific qPCR assays (Figs. 4 and S5).

In ungrazed soils, the copy numbers of *pmoA* genes in all ^13^C-spiked microcosms peaked in heavy fractions (1.738–1.778 g ml^−1^), while those in the presence of ^12^CH_4_ were detected in fractions with lower buoyant densities (between 1.680 and 1.738 g ml^−1^) (Fig. 4a–c). In grazed soils, a shift of DNA buoyant density also occurred towards the heavy fractions (1.732–1.770 g ml^−1^) in microcosms with ^13^CH_4_ under U0 and U20 treatments, but to a lesser extent than in the ungrazed soils. In addition, the distributions of *pmoA* gene abundance in microcosms with ^12^CH_4_ and microcosms under U100 + ^13^CH_4_ treatments were mainly in lighter fractions (1.698–1.732 g ml^−1^) with no peak reported in the heavy fractions (Fig. 4f–h). Sequencing of the V3–V4 region of the 16S rRNA gene generated 1,124,000 and 1,088,000 high-quality reads in ungrazed and grazed soils, respectively (Table S4). The labeling of MOB was further supported by distribution of relative abundance of methanotrophs across the whole buoyant density (Fig. S6).

The ^13^C-labeling of the AOB populations was stronger in the U100 than U20 treatments in both ungrazed and grazed soils irrespectively of methane addition treatment (Fig. 4d, e and i, j). For the ^12^CO_2_ controls (U20 + ^12^CO_2_ + ^12^CH_4_ and U100 + ^12^CO_2_ + ^12^CH_4_), the AOB *amoA* gene relative abundance was distributed in the light fractions (1.705–1.738 g ml^−1^). A small but detectable shift of DNA buoyant density was detected in U20 + ^13^CO_2_ microcosms with the relative abundance of AOB peaking in the heavy fractions (1.732–1.742 g ml^−1^) (Fig. 4d and i). A major shift into the heavier fractions was detected for bacterial *amoA* genes in U100 + ^13^CO_2_ microcosms, with a buoyant density of 1.750 g ml^−1^ (Fig. 4e and j). In contrast, no significant difference of migration between the ^12^CO_2_ and ^13^CO_2_ treatments was observed for the archaeal *amoA* gene (Fig. S5). The relative frequency of the 16S rRNA gene sequences affiliated with ammonia oxidizers across the whole buoyant density gradient of DNA fractions suggested the similar labeling trend to that of *amoA* gene abundance in the “heavy” DNA fractions (Fig. S7). Combined with the results that AOA *amoA* genes abundances decreased during incubation (Fig. S3), suggested that AOA was not important for ammonia oxidation in the grassland soil tested. Therefore, the possible bias against *Thaumarchaeota* due to primers used in the present study exerted little effect on the results.

In both ungrazed and grazed soils, a higher proportion of 16S rRNA genes affiliating to NOB were labeled in the ^13^CO_2_ than in the ^12^CO_2_ treatments, with more labeling happening in the absence of ^13^CH_4_, apart from the grazed microcosms with highest urea addition treatment (Fig. S8).

### Diversity of active methane-oxidizing bacteria and nitrifying communities

**Fig. 5:**
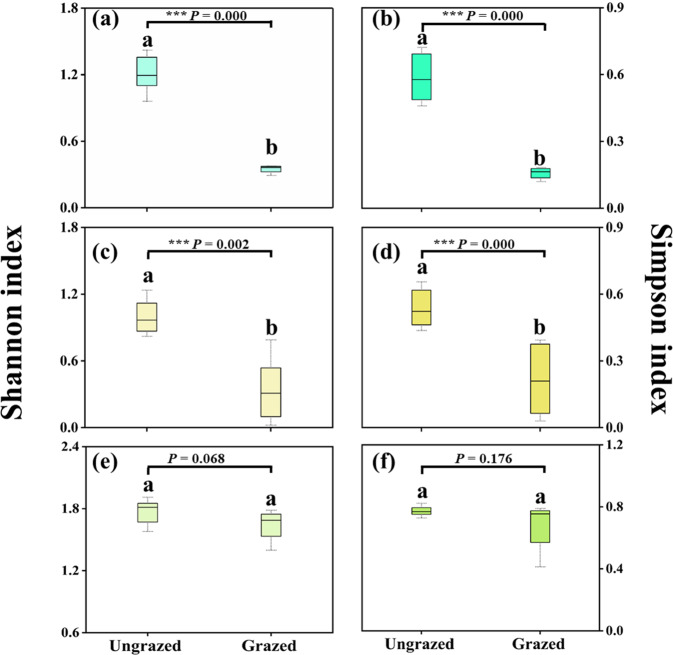
Alpha-diversity indices of active methanotrophs and nitrifiers in grassland soils. Alpha-diversity measurements of Shannon index (**a**, **c**, **e**) and Simpson index (**b**, **d**, **f**) of active MOB (**a**, **b**), AOB (**c**, **d**) and NOB (**e**, **f**) in ungrazed and grazed soils. Different letters indicate significant differences (*P* < 0.05) based on the analysis of variance.

There were obvious dissimilarities (*P* < 0.05) in diversity of active methane-oxidizing bacteria and nitrifying communities for the Shannon’s and Simpson’s indexes, respectively (Fig. 5). The Shannon’s and Simpson’s indexes of active MOB and AOB were significantly higher in ungrazed soils than those in grazed soils, while the variation of those of NOB was not significant between ungrazed and grazed soils. Grazing, therefore, significantly decreased the community diversity of active ammonia oxidizers and methanotrophs.

### Phylogenetic analysis of active MOB and AOB

The taxonomic analysis focused on the organisms present in the “heavy fractions” in the ^13^CO_2_/^13^CH_4_ microcosms, as these AOB, NOB and MOB were actively growing (Figs. 4; S5–S8).

**Fig. 6:**
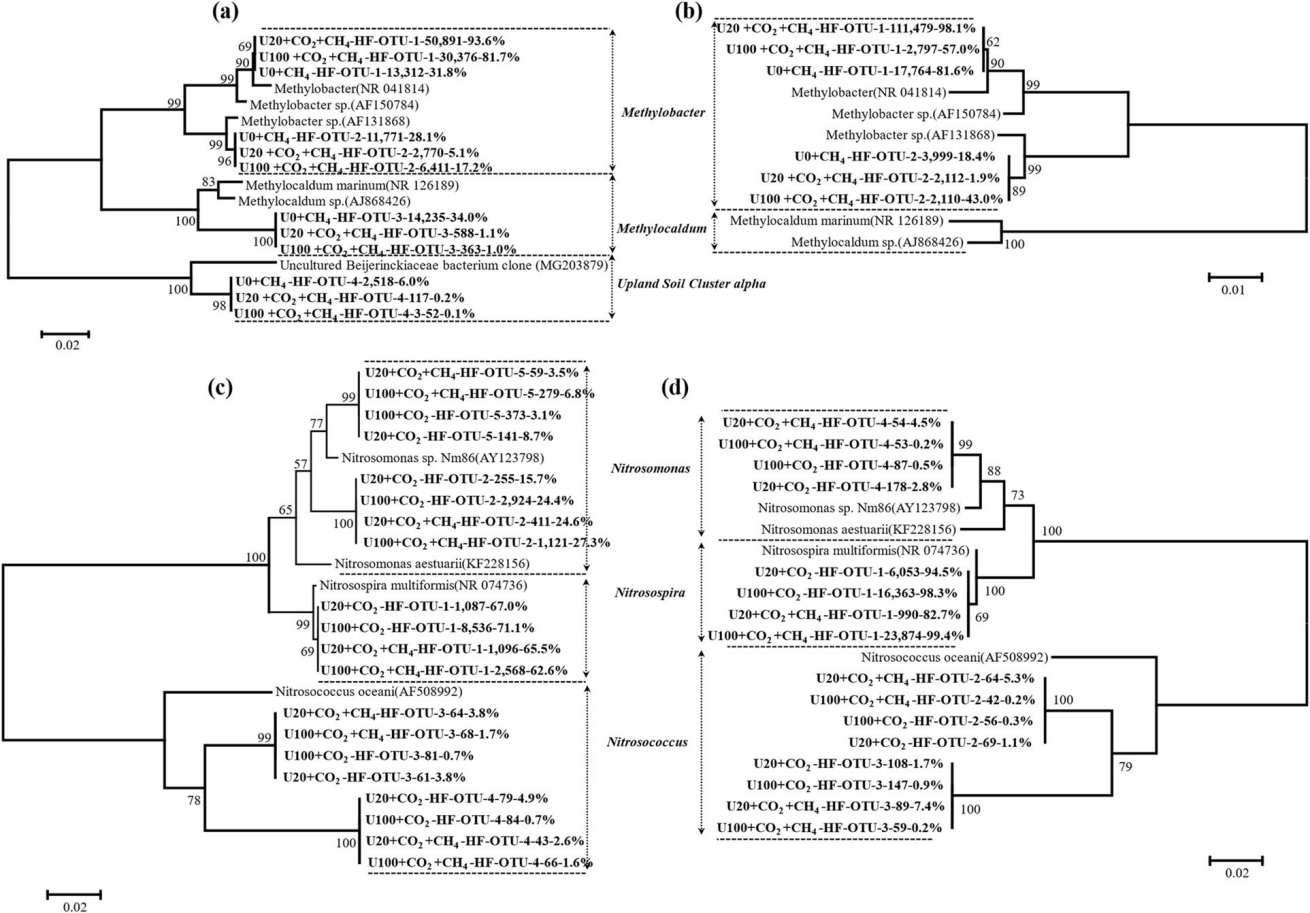
Phylogenetic tree of the ^13^C-labeled 16S rRNA genes affiliated with MOB and AOB from the labeled microcosms after incubation for 21 days. Phylogenetic analysis of the 16S rRNA genes affiliated with MOB (**a**, **b**) and AOB (**c**, **d**) in ^13^C-labeled DNA. The designation “HF” indicates the ^13^C-DNA in the active fraction after the ultracentrifugation of the total DNA extract from the labeled microcosms. The designation “U0 + CH_4_-HF-OTU-1-13,312-31.8%” indicates that OTU-1 contains 13,312 reads with > 97% sequence similarity, accounting for 31.8% of the total MOB 16S rRNA gene reads in the ^13^C-DNA from the CH_4_-treated soil microcosms. The scale bars represent 1% nucleic acid sequences divergence for the 16SrRNA genes in ungrazed (**a**) and grazed (**b**) soils, respectively. The designation “HF” indicates the ^13^C-DNA in the active fraction after the ultracentrifugation of the total DNA extract from the labeled microcosms. The designation “U20 + CO_2_-HF-OTU-1-1,087-67.0%” indicates that OTU-1 contains 1087 reads with > 97% sequence similarity, accounting for 67.0% of the total bacterial AOB 16S rRNA gene reads in the ^13^C-DNA from the U20-treated soil microcosms. The designation “U20 + CO_2_ + CH_4_-HF-OTU-1-1,096-65.5%” indicates that OTU-1 contains 1096 reads with > 97% sequence similarity, accounting for 65.5% of the total bacterial AOB 16S rRNA gene reads in the ^13^C-DNA from the U20 + CO_2_ + CH_4_-treated soil microcosms. The scale bars represent 2% nucleic acid sequences divergence for the 16SrRNA genes in ungrazed (**c**) and grazed (**d**) soils, respectively.

The majority of ^13^C-labeled MOB in ungrazed soils mainly belonged to *Methylobacter*, with some *Methylocaldum* and USCα, and AOB mainly grouped with *Nitrosospira*, with some *Nitrosomonas*, and *Nitrosococcus* (Figs. 6a, c and S9). By contrast, the ^13^C-labeled MOB and AOB in grazed soils were almost exclusively affiliated with *Methylobacter* and *Nitrosospira*, respectively (Figs. 6b, d and S9). In ungrazed soils, over 98% of active MOB sequences were derived from *Methylobacter* species in low and high urea amended soils, while 59.9% of active MOB were affiliated with *Methylobacter* in no urea treated soils (Fig. 6a).

The active AOB community grouped with *Nitrosospira* was significantly decreased from 67.0% and 71.1% in low and high urea treated soils without methane addition, respectively, to 65.5% and 62.6% in microcosms with methane addition (Fig. 6c). In stark contrast, the active AOB classified into *Nitrosomonas* was significantly increased by methane addition in both low and high N soils. Irrespective of methane addition, the active AOB community classifying as *Nitrosococcus* was suppressed by high relative to low urea addition.

### Absolute abundance of different active microbial genera affiliated with MOB and AOB

**Fig. 7:**
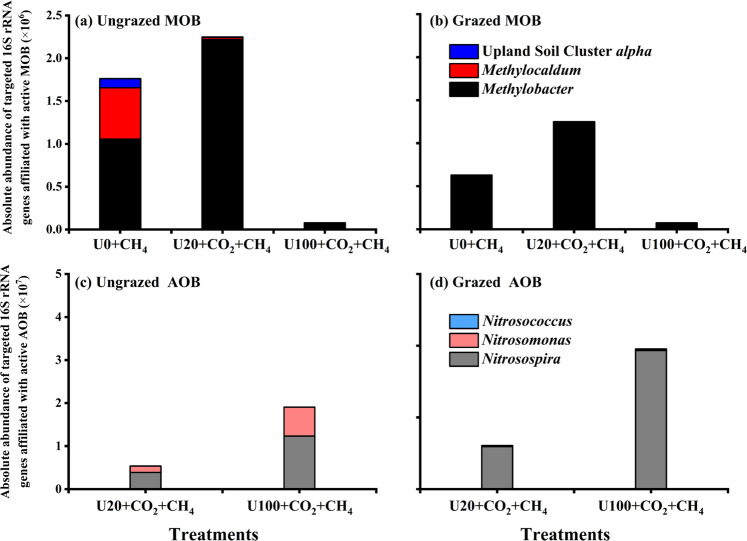
The estimate absolute abundance (EEA) of active MOB and AOB in grassland soils. Comparison of the EAA of the major active genera affiliated with MOB (**a**, **b**) and AOB (**c**, **d**) in the ungrazed (**a**, **c**) and grazed (**b**, **d**) soils after 21-day incubation with weekly urea addition of 0 (U0), 20 (U20), and 100 μg N g^−1^ (U100), and 1% methane (CH_4_).

The estimated absolute abundances (EAA) of the major active genera affiliated with active MOB and AOB were performed based on the combined analysis of specific qPCR and Illumina HiSeq sequencing analysis (Fig. 7). The EEA results showed that *Methylobacter* lineage of MOB was the dominant active MOB in the grassland ecosystems (Fig. 7a, b). The *Methylocaldum* and USCα lineages of MOB were exclusively observed in ungrazed soils without urea addition (Fig. 7a). In both ungrazed soils and in grazed soils, the addition of U20 significantly increased while U100 decreased the absolute abundance of active *Methylobacter* lineage of MOB (Fig. 7a, b). The active bacterial *amoA* genes fell within *Nitrosospira*, *Nitrosomonas,* and *Nitrosococcus*, with *Nitrosospira* accounting for the largest percentage among labeled microcosms (Fig. 7c, d). In ungrazed soils, the absolute abundance of the three lineages of AOB was higher in U100 than U20 microcosms (Fig. 7c). In grazed soils, the absolute abundance of *Nitrosospira* was 3.25-fold higher in U100 compared to U20 (Fig. 7d).

### Network description

**Fig. 8:**
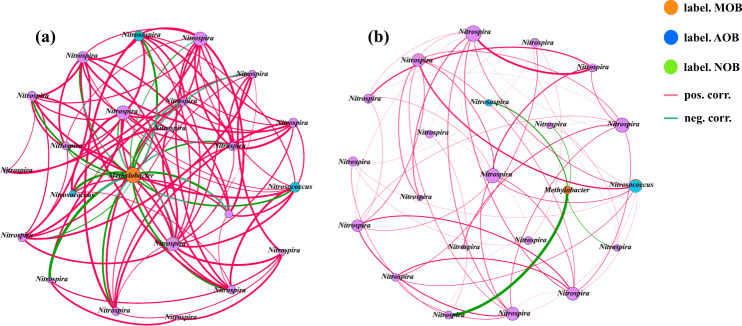
Network analysis of co-occurring active phylotypes of methanotrophs and nitrifiers in grassland soils. The co-occurring network of active MOB, AOB and NOB after 21-day incubation in the ungrazed (**a**) and grazed (**b**) soils at weekly urea addition of 20 (low) and 100 μg N g^−1^ (high) with and without 1% methane addition. Nodes represent the labeled OTUs of each of the three functional guilds, which were actively labeled with ^13^C. Isolated nodes were removed. Connecting lines (edges) correspond to significantly (*P* < 0.05) positive (red) or negative (green) correlations between nodes, where thicker lines represent stronger correlations. Labeled MOB: OTU 2 (*Methylobacter*); Labeled AOB: OTU 9 (*Nitrosospira*), OTU 466 (*Nitrosococcus*), OTU 703 (*Nitrosococcus*); Labeled NOB: OUT 5, OUT 12, OTU 55, OTU 75, OTU 126, OTU 133, OTU 191, OTU 622, OTU 816, OTU 919, OTU 2489, OTU 7401 (*Nitrospira*). The size of each node is proportional to the number of connections (i.e., degree), and the thickness of each connection between two nodes (i.e., edge) is proportional to the value of correlation coefficients. Green edges indicate positive relationships between two individual nodes, while red edges indicate negative relationships.

Network analysis based on co-occurring patterns of AOB, NOB, and MOB in “heavy fractions” in all microcosms was implemented to investigate potential relationships between the three functional guilds in ungrazed and grazed soils (Fig. 8). The resulting topological properties commonly used in network analysis in terms of the numbers of nodes and edges, average connectivity, and average clustering coefficient were detailed in Table S8. Positive and strong correlations (*P* < 0.05) were observed between active AOB and NOB phylotypes, while negative correlations (*P* < 0.05) were discerned between active MOB and the two above-mentioned nitrifiers in both ungrazed and grazed soils. In ungrazed soils, OTU 2, which fell within the *Methylobacter* and was the most abundant phylotype of active MOB (more than 80% of active MOB sequences), exhibited strong negative correlations with OTU 9, OTU 466, and OTU 703, which were affiliated with *Nitrosospira*, *Nitrosococcus*, and *Nitrosococcus*, respectively (Fig. 8a). Furthermore, in grazed soils, a strong and negative relationship was detected between *Methylobacter* and *Nitrosospira* (Fig. 8b)

## Discussion

### Grazing reduced CH_4_ uptake and increased N_2_O emissions

Grazing, associated with trampling, excreta patches and grazing grass, greatly alters plant growth, soil properties, and nutrient transformations, especially affecting soil C/N cycling [31, 43, 44]. A large number of studies investigating the effects of grazing on GHGs emissions have proposed that grazing caused a significant decline in soil CH_4_ uptake [21, 43, 45]. It was reported that soil compaction [46–48] and drought or waterlogging stress [49] decreased methane oxidation and increased methane production. The inhibition effects of grazing on CH_4_ consumption in the present study could thus be mediated by soil bulk density and moisture, which were appreciably increased and decreased under grazing, respectively, as evidenced by the strong relationships between CH_4_ uptake and bulk density (*r* = −0.962) and soil moisture (*r* = 0.966) (Table 1). Intriguingly, the effect of grazing on N_2_O emissions remains controversial. The stimulating effects of grazing on N_2_O emissions in the present study were supported by previous studies [50–52], while contrasting results were also reported [45]. However, the N_2_O emissions observed under the grazing treatment are highly dependent on the season, with most of the annual N_2_O emissions being reported from semi-arid grasslands experiencing soil freeze-thaw cycles during the spring season, while grazing decreases N_2_O emissions during the spring thaw period [45, 53], and our present measurements were taken during the grass growing season from May to October annually within 14-months. Therefore, GHGs emissions from grazed grassland soils are a result of multiple factors, including the soil properties, climate conditions, and anthropogenetic activities. The quantitative environmental impact of grazing on the global nitrogen cycle and on greenhouse gas emission needs to be fully considered and quantified to provide comprehensive recommendations for future grassland management.

**Table 1:** Physicochemical properties of the grassland soils used in this study.

Soil	pH	Soil moisture (%)	Bulk density (g cm^−3^)	Soil organic matter (g kg^−1^)	Total N (g kg^−1^)	Total C (g kg^−1^)	Olsen P (mg kg^−1^)	Available K (mg kg^−1^)	NO_3_^-^-N (mg kg^−1^)	NH_4_^+^-N (mg kg^−1^)
Ungrazed	7.19a	14.3a	1.00b	28.0a	1.63a	17.0a	5.4b	199b	42.5b	3.5b
Grazed	7.04b	7.6b	1.54a	14.7b	0.80b	9.8b	9.3a	230a	54.6a	4.9a

Values are means of triplicate. Different lowercase letters indicate significant differences between two soil types at *P* < 0.05 based on the analysis of variance.

### Communities and activity of methanotrophs and nitrifiers

The significant higher Shannon’s and Simpson’s indexes in ungrazed soils revealed that diversity of active MOB and AOB in ungrazed soils were higher than those in grazed soils. We may therefore conclude that grazing decreases the community diversity of active nitrifiers and methanotrophs in grassland soils. This could be the direct reasons why grazing suppressed CH_4_ oxidation and nitrification activity (Fig. 2).

The strong positive correlation (*r* = 0.841, *P* < 0.05) between AOB *amoA* gene abundance and nitrification activity, together with the results of DNA-SIP, indicate that AOB rather than AOA dominated microbial ammonia oxidation in the grassland soils. The predominant role of AOB in ammonia oxidation is consistent with previous studies showing that AOB generally dominates ammonia oxidation in N fertilized neutral pH agricultural soils [14,  54–57], while AOA may dominate ammonia oxidation in acidic agricultural soils [58, 59] or in unfertilized grassland soils [60]. Surprisingly, albeit the low nitrification activity, grazing appreciably increased N_2_O emissions as well as AOB *amoA* gene copy numbers and absolute abundance of active AOB affiliated with *Nitrosospira* (Figs. 1, 2, 6, 7). Apart from the soil conditions caused by grazing favored for N_2_O emissions, the significant increase of active AOB affiliated with *Nitrosospira* could also partially explain the distinct increase of N_2_O emission in grazed soils, as *Nitrosospira* were considered as the main contributors to the N_2_O emissions [61]. The active AOB in grazed soils were all affiliated with *Nitrosospira* while those in ungrazed soils were grouped into *Nitrosospira* and *Nitrosomonas* (Fig. 6). *Nitrosospira* strains were previously shown to outcompete *Nitrosomonas* in microcosms [62] while *Nitrosomonas* has a greater nitrification activity potential than *Nitrosospira* [63]. These findings might explain the unexpected increase of active AOB abundance with low nitrification activity in grazed soils. A field in situ experiment revealed that excreta depositions considerably stimulated N_2_O emissions from nitrifier denitrification dominated by AOB in grassland ecosystems [44, 64]. Combined with the results that AOB dominated nitrification, we proposed that the high N_2_O emissions in the grazed soils resulted from nitrifier denitrification possessed by *Nitrosospira* AOB in the present study. Although we failed to explore the activity of comammox in the grassland soils, comammox were found to lack NOR homologs and cannot produce N_2_O via nitrifier denitrification [65]. Meanwhile, comammox yielded N_2_O at levels that are comparable to AOA but much lower than AOB via nitrification [66]. Therefore, these results suggest that grazing tends to convert major N_2_O-generating processes from nitrification to nitrifier denitrification, which was dominated by *Nitrosospira* lineage of AOB in grassland soils. Nevertheless, more studies are needed to quantify the contribution of nitrification and nitrifier denitrification to N_2_O emissions in grazing grassland in future.

### Nitrogen-triggered relationships between CH_4_ oxidation and nitrification

The methane oxidation was increased and decreased under low and high urea addition, respectively, compared to the non-urea microcosm in both ungrazed and grazed soils (Fig. 2a). Similarly, both the *pmoA* copy numbers and the relative frequency of targeted 16S rRNA affiliated with methanotrophs were stimulated by the low rate of urea addition but suppressed by the high urea addition (Fig. 3). It has been reported that the response of the methanotrophs to different N levels is inconsistent, which is traditionally ascribed to the inherent characteristics of the methanotroph composition or the N load-tested [67]. In fact, the mineral N is essential for methanotrophs to form biomass [68]. That is why urea addition is usually observed to stimulate methane oxidation and MOB growth in soil [14–16]. The grassland soils in our present study had low fertility with low mineral N levels (Table 1), so the addition of 20 µg urea-N g^−1^ increased the abundance of MOB communities (Fig. 3), resulting in the higher methane-oxidizing activity than in the absence of urea (Fig. 2a). By contrast, the application of 100 µg urea-N g^−1^ reduced both methane oxidation and MOB growth in the grassland soils compared to no and low urea amendments (Figs. 2a and 3). High N fertilization was shown to reduce methane oxidation in some unfertilized arable land [69], while no inhibition impact was observed in paddy soils receiving annual N fertilization [14, 15]. This discrepancy might be attributed to inherent properties of the soils used (soil texture, soil N, and organic matter content), as those two studies were all carried out in paddy soils which received N fertilization annually [14, 15], whereas our present experiment was conducted in grassland soils under oligotrophic conditions (Table 1). Indeed, ammonia likely acted as the competitive inhibitor for MMO under the high urea treatment, as previously demonstrated [11]. Together, these results suggested that N was a trigger unlocking putative competition between methane and ammonia oxidation in global carbon and nitrogen cycling.

### Putative competition between active nitrifiers and MOB

This study observed negative correlations between *Methylobacter*-like MOB and *Nitrosospira* lineage of AOB, while strong and positive correlations between AOB and NOB in both ungrazed and grazed soils (Fig. 8). *Methylobacter* was the most responsive MOB in all the microcosms regardless of urea levels (Figs. 6a, b, 7a, b), indicating the competitive life strategy of *Methylobacter* [67]. Similarly, *Nitrosospira* species dominated AOB communities and were ubiquitous in all treatments (Figs. 6c, d, 7c, d). Strong positive links could be attributed to niche overlap and cross-feeding, while negative relationships could be attributed to competition and amensalism [70]. The strong negative correlations between *Methylobacter*-like MOB and *Nitrosospira* lineage of AOB suggest the putative competitive relationships between these two critical players of soil C and N cycling competing for N or even for oxygen in the soils [14]. The strong and positive correlations between AOB and NOB suggested putative syntrophic relationships between those nitrifiers, as the nitrite produced by AOB is a substrate for NOB [71].

In the ungrazed control soils, strong negative relationships occurred between *Methylobacter* and both *Nitrosospira* and *Nitrosococcus* (Fig. 8a). In the grazed soils, the putative competitive relationships between MOB and AOB exclusively occurred between *Methylobacter* and *Nitrosospira* (Fig. 8b). These results indicated that the network in ungrazed soils incorporated a substantially higher number of significant correlations than that in grazed soils. Previous study pointed out that grazing weakened the correlations between soil micro‐food webs and ecosystem functions (soil C and N mineralization) [72]. To the best of our knowledge, the present study is the first work to reveal the impact of grazing on putative competition between active methanotrophs and nitrifiers. We did not directly analyze the interactions of one group upon another, and between the taxonomic groups and process rates. In addition, some important controls (e.g., U0 + ^12^CH_4_, U0 + ^12^CO_2_, U0 + ^13^CO_2_) should be taken into consideration for further studies targeting the active methanotrophs and nitrifiers under N gradient. Nonetheless, our study unraveled the putative competitive relationships between active MOB and AOB based on the abundance, activity, composition, and network analysis of the functional genes. These results suggest that grazing decreases methane and ammonia oxidation activity, and diversity of nitrifiers as well as methanotrophs, and subsequently weakens the putative competitive relationships between methanotrophs and nitrifiers in grassland soils.

## Conclusions

In this study, we linked the activity, composition, and potential relationships between active methanotrophs and nitrifying communities using stable-isotope techniques. *Methylobacter* lineage of MOB and *Nitrosospira* lineage of AOB were the dominating active methane and ammonia oxidizing microorganisms, respectively, in ungrazed and grazed soils, and *Nitrosococcus* and *Nitrosomonas* lineages of AOB were also involved in ammonia oxidation in ungrazed soils. The diversities of active MOB and AOB communities were higher in ungrazed than in grazed soils. And the network of co-occurring active phylotypes of MOB, AOB, and NOB was also more complex in ungrazed than in grazed soils, while the methane and ammonia oxidation activities were higher in ungrazed than in grazed soils. Therefore, these results suggest that grazing decreased diversity of active microbes mediated in C/N cycling, thus suppressing both methane oxidation and nitrification activity, weakening their putative competitive relationships, and thereby increasing methane emissions. These studies will help to understand the complex biotransformation processes of C and N, which are important for development of management practices to mitigate greenhouse-gas emissions in grassland soils.

## Supplementary information

Supplementary Materials
