# Correction to: Grazing weakens competitive interactions between active methanotrophs and nitrifiers modulating greenhouse-gas emissions in grassland soils

**DOI:** 10.1038/s43705-022-00090-y

**Published:** 2022-01-24

**Authors:** Hong Pan, Haojie Feng, Yaowei Liu, Chun-Yu Lai, Yuping Zhuge, Qichun Zhang, Caixian Tang, Hongjie Di, Zhongjun Jia, Cécile Gubry-Rangin, Yong Li, Jianming Xu

**Affiliations:** 1 Institute of Soil and Water Resources and Environmental Science, College of Environmental and Resource Sciences, Zhejiang Provincial Key Laboratory of Agricultural Resources and Environment, Zhejiang University, Hangzhou, 310058 China; 2 National Engineering Laboratory for Efficient Utilization of Soil and Fertilizer Resources, College of Resources and Environment, Shandong Agricultural University, Daizong Road, Tai’an City, Shandong 271018 China; 3 Advanced Water Management Centre, The University of Queensland, St Lucia, QLD 4072 Australia; 4 Department of Animal, Plant and Soil Sciences, Centre for AgriBioscience, La Trobe University, Bundoora, VIC 3086 Australia; 5 State Key Laboratory of Soil and Sustainable Agriculture, Institute of Soil Science, Chinese Academy of Sciences, Nanjing, 210008 China; 6 School of Biological Sciences, University of Aberdeen, Aberdeen, AB24 3UU UK

**Keywords:** Soil microbiology, Biogeochemistry

Correction to: *ISME Communications* 10.1038/s43705-021-00068-2, published online 9 December 2021

The “CH_4_ emissions” should be “CH_4_ uptake” in the sentence of “Grazing significantly decreases CH_4_ emissions while it increases N_2_O emissions basing on 14-month in situ measurement”

The correct sentence should be read:

Grazing significantly decreases CH_4_ uptake while it increases N_2_O emissions basing on 14-month in situ measurement

The original article has been revised.

