# Supplementary material for: Grazing weakens competitive interactions between active methanotrophs and nitrifiers modulating greenhouse-gas emissions in grassland soils
Source: ISME Commun. 2021 Dec 9;1:74. doi: 10.1038/s43705-021-00068-2 (PMC9723554; doi:10.1038/s43705-021-00068-2)
Supplement: Supplementary file 1 — Supplementary Materials [file 43705_2021_68_MOESM1_ESM.docx]

***Supporting Information (SI) for:***

**Grazing weakens putative competitions between active methanotrophs and nitrifiers modulating greenhouse-gas emissions in grassland soils**

Hong Pan^a,b,1^, Haojie Feng^b,1^, Yaowei Liu^a^, Chun-Yu Lai^c^, Yuping Zhuge^b^, Qichun Zhang^a^, Caixian Tang^d^, Hongjie Di^a^, Zhongjun Jia^e^, Cécile Gubry-Rangin^f,*^, Yong Li^a,*^, Jianming Xu^a^

^a^ Institute of Soil and Water Resources and Environmental Science, College of Environmental and Resource Sciences, Zhejiang Provincial Key Laboratory of Agricultural Resources and Environment, Zhejiang University, Hangzhou 310058, China

^b^ National Engineering Laboratory for Efficient Utilization of Soil and Fertilizer Resources, College of Resources and Environment, Shandong Agricultural University, Daizong Road, Tai’an City, Shandong 271018, China

^c^Advanced Water Management Centre, The University of Queensland, St Lucia, Queensland 4072, Australia

^d^ Department of Animal, Plant and Soil Sciences, Centre for AgriBioscience, La Trobe University, Bundoora, VIC 3086, Australia

^e^ State Key Laboratory of Soil and Sustainable Agriculture, Institute of Soil Science, Chinese Academy of Sciences, Nanjing, 210008, China

^f^ School of Biological Sciences, University of Aberdeen, Aberdeen, AB24 3UU, United Kingdom

^*^ Corresponding authors: Dr. Yong Li ([liyongcn@zju.edu.cn](mailto:liyongcn@zju.edu.cn)) and Dr. Cécile Gubry-Rangin (c.rangin@abdn.ac.uk)

^1^ These authors contributed equally to this work.

29 text pages, 8 tables, 9 figures

**Results**

**Compositions of the soil methanotrophs and nitrifying communities**

The HiSeq sequencing was carried out using the total DNA extracted from soil microcosms on day 0 and day 21. Approximately 2.2 million high-quality sequence reads were obtained (Table S4). HiSeq-sequencing of 16S rRNA genes generated about 169,000 and 191,000 high-quality sequence reads affiliated with MOB in ungrazed and grazed soils, respectively, while about 20,000 and 50,000 AOB gene sequence reads were yielded in ungrazed and grazed soils, respectively. In addition, HiSeq was also conducted using the amplicons synthesized with a universal 515F-907R primer of the fractions (3-12) of the labeled (^13^CO_2_ and ^13^CH_4_) and control (^12^CO_2_ and ^12^CH_4_) microcosms at Day 21 (Table S5). Approximately 2.8 million and 3.3 million high-quality reads were obtained in the fractionated DNA for ungrazed and grazed soils, respectively.

The 16S rRNA genes affiliated with MOB were selected for phylogenetic analysis from the total DNA and were phylogenetically assigned to *Methylobacter*, *Methylocaldum*, Upland soil clusters (alpha and gamma in the present study) and *Methylocystis* on Days 0 and 21 (Figs. S8a). In ungrazed soils, over 94% of MOB sequences were derived from *Methylobacter* species (Fig. S9a). The application of methane without urea addition led to an increase in relative abundance of *Methylocaldum* and Upland soil cluster alpha (USCα) from 2.1% and 1.6% on Day 0 to 34.0% and 6.0% on Day 21, respectively (Fig. S9a). In stark contrast, up to 98% of active MOB was affiliated with *Methylobacter* in urea-amended microcosms on Day 21 (Fig. 6a; Fig. S9a). In grazed soils, over 99% of the *pmoA* sequences were detected exclusively as *Methylobacter* in microcosms irrespective of urea amendment or sampling time (Fig. 6b; Fig. S9a).

The majority of the AOB sequences were affiliated with *Nitrosococcus*, *Nitrosospira* and *Nitrosomonas* in the grassland soils (Fig. S9b). The phylogenetic analysis unraveled a clear shift of AOB community pattern over time (Fig. S9b). In ungrazed soils, 66.7% of AOB community was phylogenetically assigned to the genus *Nitrosococcus* on Day 0, while the active bacterial *amoA* genes were classified into *Nitrosospira* after 21-day incubation (Fig. 6c; Fig. S9b). Methane addition increased the proportion of *Nitrosomonas* lineage from 24.4% and 27.5% on day 0 to 28.1% and 34.1% on day 21 in the U20 and U100-treated microcosms, respectively. Irrespective of methane addition, the active AOB community fell into *Nitrosococcus* was clearly suppressed by U100 relative to U20 (Fig. 6c; Fig. S9b). In grazed soils, 55% of AOB community was divided into *Nitrosospira* and *Nitrosococcus*, only a minority of OTUs were classified into *Nitrosomonas* on Day 0 (Fig. S9b). However, the active AOB sequences were mainly divided into *Nitrosospira*, with a few AOB sequences being grouped into *Nitrosomonas* and *Nitrosococcus* on Day 21 in U20-treated microcosms. *Nitrosospira* lineage of AOB accounted for over 98% of active AOB in U100-treated microcosms after 21 days of incubation (Fig. 6c; Fig. S9b).

**Supplementary Tables S1–S8**

**Table S1** Schematic overview of the amendments made to the microcosms.

**Table S2** Schematic overview of CH_4_ addition over the course of 21 days of incubation.

**Table S3** Primers and conditions used in this study.

**Table S4** Sequencing summary of the total microbial communities in SIP microcosms using the universal primers 515F-907R of the total 16S rRNA genes.

**Table S5** Sequencing summary of the total microbial communities in the fractionated DNA by isopycnic centrifugation of total DNA extracted from SIP microcosms using the universal primers 515F-907R of the total 16S rRNA genes.

**Table S6** Changes of quantitative distribution of methylotrophs in soil microcosms over an incubation period of 21 days in the ungrazed and grazed soils at weekly urea addition of 0 (U0), 20 (U20) and 100 μg N g^-1^ (U100) with 1% methane (+CH_4_). The *pmoA* gene copy numbers in total DNA, relative frequencies of targeted 16S rRNA genes affiliated with MOB in total reads (%) and relative frequency of targeted 16S rRNA genes affiliated with methanol oxidizing bacteria in total reads (%) in the ungrazed and grazed soils after 21-day incubation. There are 3 replicates for the U0+CH_4_ treatments, and 6 replicates for the U20 and U100 treatments (^12^C + ^13^C-labeled treatments).

**Table S7** Changes of quantitative distribution of AOB *amoA* genes in soil microcosms over an incubation period of 21 days in the ungrazed and grazed soils at weekly urea addition of 20 (U20) and 100 μg N g^-1^ (U100) in presence or absence of methane addition (1% CH_4_). The *amoA* gene copy numbers in total DNA, relative frequencies of targeted 16S rRNA genes affiliated with AOB in total reads (%) in the ungrazed and grazed soils after 21-day incubation. There are 6 replicates for the U20 and U100 treatments (^12^C + ^13^C-labeled treatments).

**Table S8** Network topological characteristics calculated by Network Analyzer tool in

R 3.6.0

**Supplementary Figures S1-S9**

**Fig. S1** Dynamic monitoring of methane consumption in soil microcosms over an incubation period of 21 days. The y-axis is expressed as the percentage of concentrations of methane left in the microcosms relative to the initial methane concentrations.

**Fig. S2** Changes in the soil NO_3_^-^-N (a, b) and NH_4_^+^-N (c, d) concentrations over a 21-day incubation period in the SIP microcosms. The error bars represent the standard errors of the mean of triplicate microcosms, while for the soil microcosms of Urea+CO_2_+CH_4_ treatments 6 replicates were used including both ^12^C-control and ^13^C-labeled treatments.

**Fig. S3** Changes of quantitative distribution of AOA *amoA* genes in soil microcosms over an incubation period of 21 days in ungrazed (a, c) and grazed (b, d) soils. The *amoA* gene copy numbers in total DNA from microcosms after incubation for 21 days in ungrazed (a) and grazed (b) soils; Relative frequencies of targeted 16S rRNA genes affiliated with AOA in total reads (%) in ungrazed (c) and grazed (d) soils. The error bars represent standard errors of 6 replicates including both ^12^C-control and ^13^C-labeled treatments.

**Fig. S4** The relative abundances of NOB in ungrazed (a) and grazed (b) soils determined by high-throughput fingerprinting of 16S rRNA genes. The error bars represent the standard errors of the mean of triplicate microcosms, while for the soil microcosms of Urea+CO_2_+CH_4_ treatments 6 replicates were used including both ^12^C-control and ^13^C-labeled treatments.

**Fig. S5** Quantitative distribution of archaeal *amoA* genes across the entire buoyant density gradient of the fractionated DNA from the ungrazed (a) and grazed (b) soils at weekly urea addition of 0 (U0), 20 (U20) and 100 μg N g^-1^ (U100), in presence or absence of methane addition (1% CH_4_) after incubation for 21 days. The normalized data are shown as the ratio of the gene copy number in each fraction to the maximum quantities in each treatment. The dotted and plain lines represent samples in ungrazed and grazed soils, respectively.

**Fig. S6** The relative frequency of the targeted 16S rRNA genes affiliated with methylotrophs in total reads. The ratios were calculated by targeted 16S rRNA gene reads affiliated with MOB to the total microbial 16S rRNA gene reads across the entire buoyant density gradient of the fractionated DNA from soil microcosms after incubation for 21 days in ungrazed (a-c) and grazed (d-f) soils.

**Fig. S7** The relative frequency of the targeted 16S rRNA genes affiliated with nitrifiers in total reads. The ratios were calculated by targeted 16S rRNA gene reads affiliated with nitrifiers to the total microbial 16S rRNA gene reads across the entire buoyant density gradient of the fractionated DNA from soil microcosms after incubation for 21 days in ungrazed (a-c) and grazed (d-f) soils.

**Fig. S8** The relative frequency of the targeted 16S rRNA genes affiliated with NOB in total reads. The ratios were calculated by targeted 16S rRNA gene reads affiliated with NOB to the total microbial 16S rRNA gene reads across the entire buoyant density gradient of the fractionated DNA from soil microcosms after incubation for 21 days in ungrazed (a, b) and grazed (c, d) soils.

**Fig. S9** Proportional changes of methanotroph (a) and AOB (b) phylotypes in SIP microcosms after an incubation period of 21 days in Ungrazed and Grazed soils. The changes in active MOB and AOB composition were analyzed by target 16S rRNA genes in the active DNA from labeled microcosms at day 21.

**Supplementary Tables**

**Table S1** Schematic overview of the amendments made to the microcosms

| **Treatment** | **CH_4_**  **(v/v)** | **Urea**  **(μg g^-1^)** | **CO_2_**  **(v/v)** |
| --- | --- | --- | --- |
| **U0 + ^13^C-CH_4_** | **1%** | **-** | **-** |
| **U20 + ^13^C-CO_2_** | **-** | **20** | **5%** |
| **U100 + ^13^C-CO_2_** | **-** | **100** | **5%** |
| **U20 + ^13^C-CO_2_ + ^13^C-CH_4_** | **1%** | **20** | **5%** |
| **U100 + ^13^C-CO_2_ + ^13^C-CH_4_** | **1%** | **100** | **5%** |
| **U20 + ^12^C-CO_2_ + ^12^C-CH_4_** | **1%** | **20** | **5%** |
| **U100 + ^12^C-CO_2_ + ^12^C-CH_4_** | **1%** | **100** | **5%** |

**Table** **S2** Schematic overview of CH_4_ addition over the course of 21 days of incubation

| Ungrazed | ^13^CH_4_ | U20+^13^CO_2_+^13^CH_4_ | U100+^13^CO_2_+^13^CH_4_ | U20+^12^CO_2_+^12^CH_4_ | U100+^12^CO_2_+^12^CH_4_ |  |  |  |  |  |  |
| --- | --- | --- | --- | --- | --- | --- | --- | --- | --- | --- | --- |
| Day-0 | 10000 | 10000 | 10000 | 10000 | 10000 |  |  |  |  |  |  |
| Day-5 | - | - | - | - | - |  |  |  |  |  |  |
| Day-7 | 10000 | 10000 | 10000 | 10000 | 10000 |  |  |  |  |  |  |
| Day-11 | 7483 | 10084 | 10076 | 10081 | 10075 |  |  |  |  |  |  |
| Day-13 | 8592 | 10080 | 3999 | 10075 | 6926 |  |  |  |  |  |  |
| Day-14 | 10000 | 10000 | 10000 | 10000 | 10000 |  |  |  |  |  |  |
| Day-16 | 7635 | 9428 | 2634 | 8716 | 5946 |  |  |  |  |  |  |
| Day-19 | 8669 | 10084 | 583 | 10062 | 1518 |  |  |  |  |  |  |

| Grazed | ^13^CH_4_ | U20+^13^CO_2_+^13^CH_4_ | U100+^13^CO_2_+^13^CH_4_ | U20+^12^CO_2_+^12^CH_4_ | U100+^12^CO_2_+^12^CH_4_ |
| --- | --- | --- | --- | --- | --- |
| Day-0 | 10000 | 10000 | 10000 | 10000 | 10000 |
| Day-5 | - | - | - | - | - |
| Day-7 | 10000 | 10000 | 10000 | 10000 | 10000 |
| Day-11 | 639 | 3110 | 753 | 1278 | 456 |
| Day-13 | 466 | 1479 | 487 | 662 | 632 |
| Day-14 | 10000 | 10000 | 10000 | 10000 | 10000 |
| Day-16 | 2445 | 7376 | 2079 | 1537 | 1000 |
| Day-19 | 4084 | 9661 | 483 | 559 | 413 |

**Table S3** Primers and conditions used in this study

| Primer Name | Primer sequence (5′-3′) | Target gene | Thermal Profile | Reference |
| --- | --- | --- | --- | --- |
| Arch-amoAF | STA ATG GTC TGG CTT AGA CG | archaeal *amoA* gene | 95℃/2m;40cycles of 95℃/20s,55 ℃/20s,72℃/30s | [1] |
| Arch-amoAR | GCG GCC ATC CAT CTG TAT GT |  |  |  |
| amoA-1F | GGG GTT TCT ACT GGT GGT | bacterial *amoA* gene | 95℃/2m;40cycles of 95℃/20s,57℃/30s,72℃/30s | [2] |
| amoA-2R | CCC CTC KGS AAA GCC TTC TTC |  |  |  |
| A189F | GGNGACTGGGACTTCTGG | *pmoA* gene | 94°C/2m; 40 cycles of 94°C/20s, 60°C/45s, 72°C/30s | [3] |
| mb661r | CCGGMGCAACGTCYTTACC |  |  |  |
| 515F | GTG CCA GCM GCC GCG G | universal 16S rRNA genes | 95°C/ 3m; 32 cycles of (95°C/30s, 55°C/30s, 72°C/30s); 72°C/10m | [4] |
| 907R | CCG TCA ATT CMT TTR AGT TT |  |  |  |

**Table S4** Sequencing summary of the total microbial communities in SIP microcosms using the universal primers 515F-907R of the total 16S rRNA genes

| Treatment | | Sequencing reads number | | | | | | |
| --- | --- | --- | --- | --- | --- | --- | --- | --- |
|  |  | High-quality read number | | | | | | |
|  |  | ^13^CH_4_ | U20+^13^CO_2_ | U100+^13^CO_2_ | U20+^13^CH_4_+^13^CO_2_ | U100+^13^CH_4_+^13^CO_2_ | U20+^12^CH_4_+^12^CO_2_ | U100+^12^CH_4_+^12^CO_2_ |
| Ungrazed | 0d R1 | 45518 |  |  |  |  |  |  |
|  | 0d R2 | 43233 |  |  |  |  |  |  |
|  | 0d R3 | 57819 |  |  |  |  |  |  |
|  | 21d R1 | 42288 | 43327 | 45296 | 53434 | 49262 | 59346 | 65259 |
|  | 21d R2 | 60129 | 47041 | 40852 | 51881 | 46225 | 57235 | 46599 |
|  | 21d R3 | 39086 | 40003 | 34677 | 40132 | 35700 | 38872 | 41141 |
| Grazed | 0d R1 | 33229 |  |  |  |  |  |  |
|  | 0d R2 | 39485 |  |  |  |  |  |  |
|  | 0d R3 | 31235 |  |  |  |  |  |  |
|  | 21d R1 | 34488 | 35799 | 47262 | 57025 | 40182 | 52709 | 64031 |
|  | 21d R2 | 35974 | 34234 | 64558 | 47095 | 49879 | 57078 | 54748 |
|  | 21d R3 | 51964 | 32239 | 45399 | 49055 | 45380 | 42392 | 43465 |
|  | Average | 46110 |  |  |  |  |  |  |
|  | Total | 2213260 |  |  |  |  |  |  |

**Table S5** Sequencing summary of the total microbial communities in the fractionated DNA by isopycnic centrifugation of total DNA extracted from SIP microcosms using the universal primers 515F-907R of the total 16S rRNA genes.

| Soil | DNA gradient fraction | High-quality reads number | | | | | | |
| --- | --- | --- | --- | --- | --- | --- | --- | --- |
|  |  | ^13^CH_4_ | U20+^13^CO_2_ | U100+^13^CO_2_ | U20+^13^CH_4_+^13^CO_2_ | U100+^13^CH_4_+^13^CO_2_ | U20+^12^CH_4_+^12^CO_2_ | U100+^12^CH_4_+^12^CO_2_ |
| Ungrazed | Fraction-3 | 44292 | 60580 | 37427 | 50165 | 39182 | 63632 | 40859 |
|  | Fraction-4 | 42797 | 36248 | 41660 | 37791 | 57580 | 67102 | 25384 |
|  | Fraction-5 | 33919 | 55720 | 47341 | 49475 | 42038 | 48879 | 18232 |
|  | Fraction-6 | 36410 | 39357 | 36105 | 42035 | 42159 | 42894 | 31905 |
|  | Fraction-7 | 38157 | 38842 | 36777 | 37399 | 42019 | 41324 | 35101 |
|  | Fraction-8 | 35177 | 36106 | 31362 | 32787 | 36026 | 35641 | 38265 |
|  | Fraction-9 | 33255 | 37411 | 38503 | 42327 | 29443 | 38285 | 36105 |
|  | Fraction-10 | 39249 | 49152 | 41439 | 39082 | 37099 | 41936 | 38289 |
|  | Fraction-11 | 42432 | 49578 | 38951 | 41589 | 46609 | 53103 | 45421 |
|  | Fraction-12 | 46636 | 37438 | 39185 | 28267 | 41396 | 37358 | 37473 |
|  | Average | 39232 | 44043 | 38875 | 40092 | 41355 | 47015 | 34703 |
|  | Subtotal | 392324 | 440432 | 388750 | 400917 | 413551 | 470154 | 347034 |
|  | Total | 2853162 |  |  |  |  |  |  |
| Grazed | Fraction-3 | 47483 | 38194 | 33240 | 48463 | 39064 | 49469 | 35845 |
|  | Fraction-4 | 43140 | 44851 | 41191 | 61325 | 47517 | 53102 | 52582 |
|  | Fraction-5 | 42684 | 46061 | 39387 | 70846 | 39559 | 26299 | 33360 |
|  | Fraction-6 | 42130 | 41302 | 35844 | 64661 | 49269 | 53595 | 46357 |
|  | Fraction-7 | 51667 | 56283 | 32585 | 46414 | 60178 | 49340 | 43041 |
|  | Fraction-8 | 53095 | 40925 | 23430 | 57577 | 58515 | 48993 | 48418 |
|  | Fraction-9 | 55519 | 37680 | 26456 | 50618 | 59355 | 64467 | 59392 |
|  | Fraction-10 | 52313 | 46579 | 52372 | 60694 | 59523 | 69031 | 61886 |
|  | Fraction-11 | 54123 | 42580 | 57608 | 44366 | 41885 | 66637 | 50222 |
|  | Fraction-12 | 53973 | 40637 | 38676 | 39905 | 54736 | 58530 | 4465 |
|  | Average | 49613 | 43509 | 38079 | 54487 | 50960 | 53946 | 43557 |
|  | Subtotal | 496127 | 435092 | 380789 | 544869 | 509601 | 539463 | 435568 |
|  | Total | 3341509 |  |  |  |  |  |  |

**Table S6** Changes of quantitative distribution of methylotrophs in soil microcosms over an incubation period of 21 days in the ungrazed and grazed soils at weekly urea addition of 0 (U0), 20 (U20) and 100 μg N g^-1^ (U100) with 1% methane (+CH_4_). The *pmoA* gene copy numbers in total DNA, relative frequencies of targeted 16S rRNA genes affiliated with MOB in total reads (%) and relative frequency of targeted 16S rRNA genes affiliated with methanol oxidizing bacteria in total reads (%) in the ungrazed and grazed soils after 21-day incubation. There are 3 replicates for the U0+CH_4_ treatments, and 6 replicates for the U20 and U100 treatments (^12^C + ^13^C-labeled treatments).

| Soil | Ungrazed | | | Grazed | |
| --- | --- | --- | --- | --- | --- |
|  | 0d | 21d | 0d | | 21d |
| The *pmoA* gene copy numbers in total DNA | | | | | |
| U0+CH_4_ | 2.02×10^5^ | 1.76×10^6^ | 1.16×10^5^ | | 6.32×10^5^ |
| U20+CO_2_+CH_4_ | 2.02×10^5^ | 2.34×10^6^ | 1.22×10^5^ | | 1.25×10^6^ |
| U100+CO_2_+CH_4_ | 2.02×10^5^ | 2.37×10^5^ | 1.06×10^5^ | | 7.43×10^4^ |
| The relative frequencies of targeted 16S rRNA genes affiliated with MOB in total reads (%) | | | | | |
| U0+CH_4_ | 2.138 | 3.580 | 0.534 | | 1.643 |
| U20+CO_2_+CH_4_ | 2.138 | 13.153 | 0.534 | | 4.109 |
| U100+CO_2_+CH_4_ | 2.138 | 1.837 | 0.534 | | 0.837 |
| The relative frequencies of targeted 16S rRNA genes affiliated with methanol oxidizing bacteria in total reads (%) | | | | | |
| U0+CH_4_ | 0.432 | 0.834 | 0.060 | | 0.267 |
| U20+CO_2_+CH_4_ | 0.432 | 0.884 | 0.060 | | 0.725 |
| U100+CO_2_+CH_4_ | 0.432 | 0.267 | 0.060 | | 0.103 |

**Table S7** Changes of quantitative distribution of AOB *amoA* genes in soil microcosms over an incubation period of 21 days in the ungrazed and grazed soils at weekly urea addition of 20 (U20) and 100 μg N g^-1^ (U100) in presence or absence of methane addition (1% CH_4_). The *amoA* gene copy numbers in total DNA, relative frequencies of targeted 16S rRNA genes affiliated with AOB in total reads (%) in the ungrazed and grazed soils after 21-day incubation. There are 6 replicates for the U20 and U100 treatments (^12^C + ^13^C-labeled treatments).

| Soil | Ungrazed | | Grazed | |
| --- | --- | --- | --- | --- |
|  | 0d | 21d | 0d | 21d |
| The *amoA* gene copy numbers in total DNA | | | | |
| U20+CO_2_ | 3.44×106 | 5.24×106 | 7.33×106 | 1.08×107 |
| U20+CO_2_+CH_4_ | 2.94×106 | 5.43×106 | 5.83×106 | 1.21×107 |
| U100+CO_2_ | 5.30×106 | 2.01×107 | 7.57×106 | 3.98×107 |
| U100+CO_2_+CH_4_ | 4.80×106 | 1.97×107 | 6.40×106 | 3.91×107 |
| The relative frequencies of targeted 16S rRNA genes affiliated with AOB in total reads (%) | | | | |
| U20+CO_2_ | 0.065 | 0.072 | 0.100 | 0.150 |
| U20+CO_2_+CH_4_ | 0.065 | 0.081 | 0.100 | 0.189 |
| U100+CO_2_ | 0.065 | 0.267 | 0.100 | 0.481 |
| U100+CO_2_+CH_4_ | 0.065 | 0.230 | 0.100 | 0.555 |

**Table S8** Network topological characteristics calculated by Network Analyzer tool in R 3.6.0.

|  | Ungrazed soil | Grazed soil |
| --- | --- | --- |
| Number of nodes | 23 | 22 |
| Number of edges | 97 | 59 |
| Network diameter | 4 | 7 |
| Network density | 0.383 | 0.255 |
| Average clustering coefficient | 0.728 | 0.75 |
| Average path length | 1.775 | 2.861 |
| Modularity | 0.217 | 0.391 |

**Supplementary Figures**


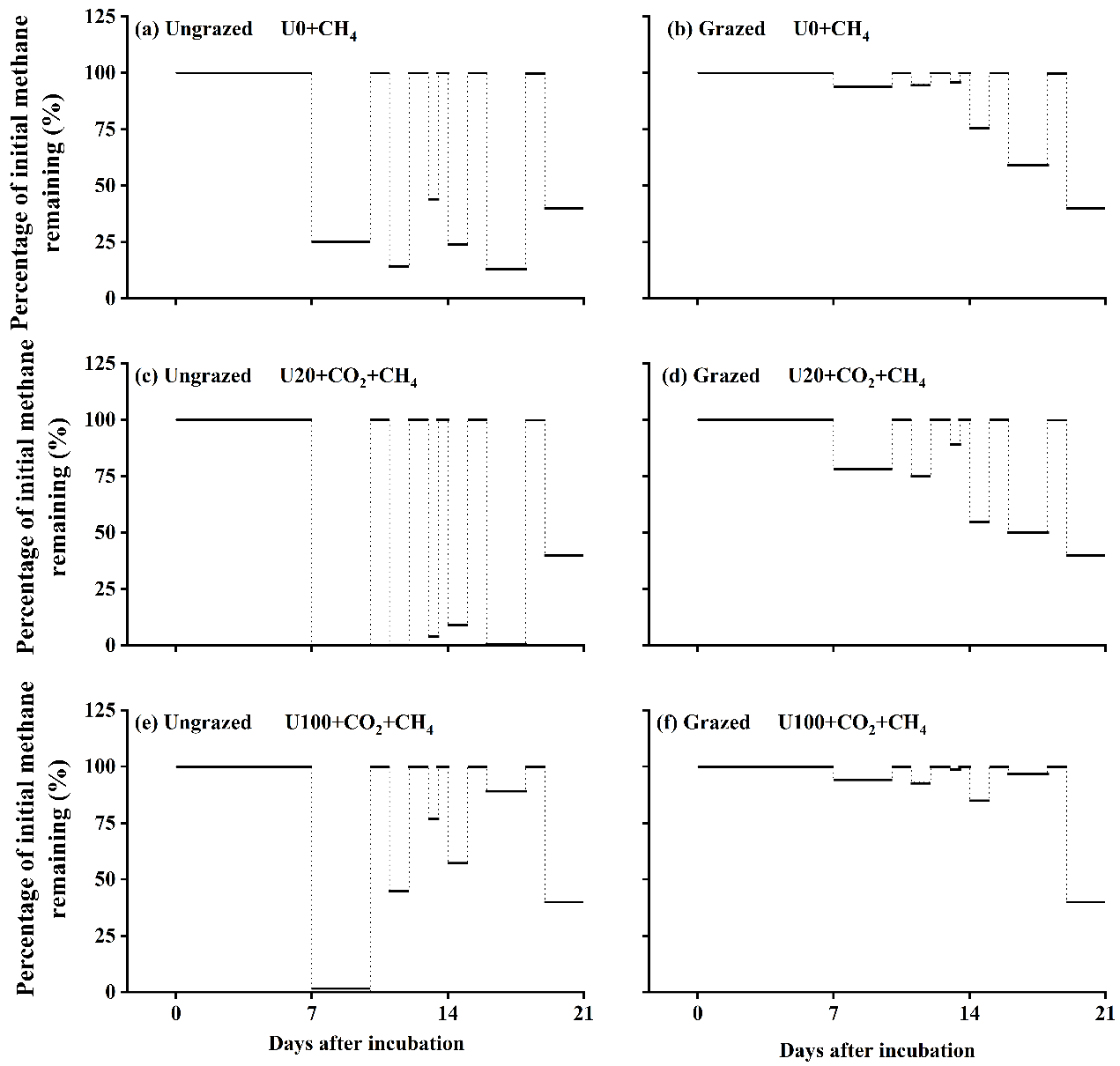


**Fig. S1** Dynamic monitoring of methane consumption in soil microcosms over an incubation period of 21 days. The y-axis is expressed as the percentage of concentrations of methane left in the microcosms relative to the initial methane concentrations.


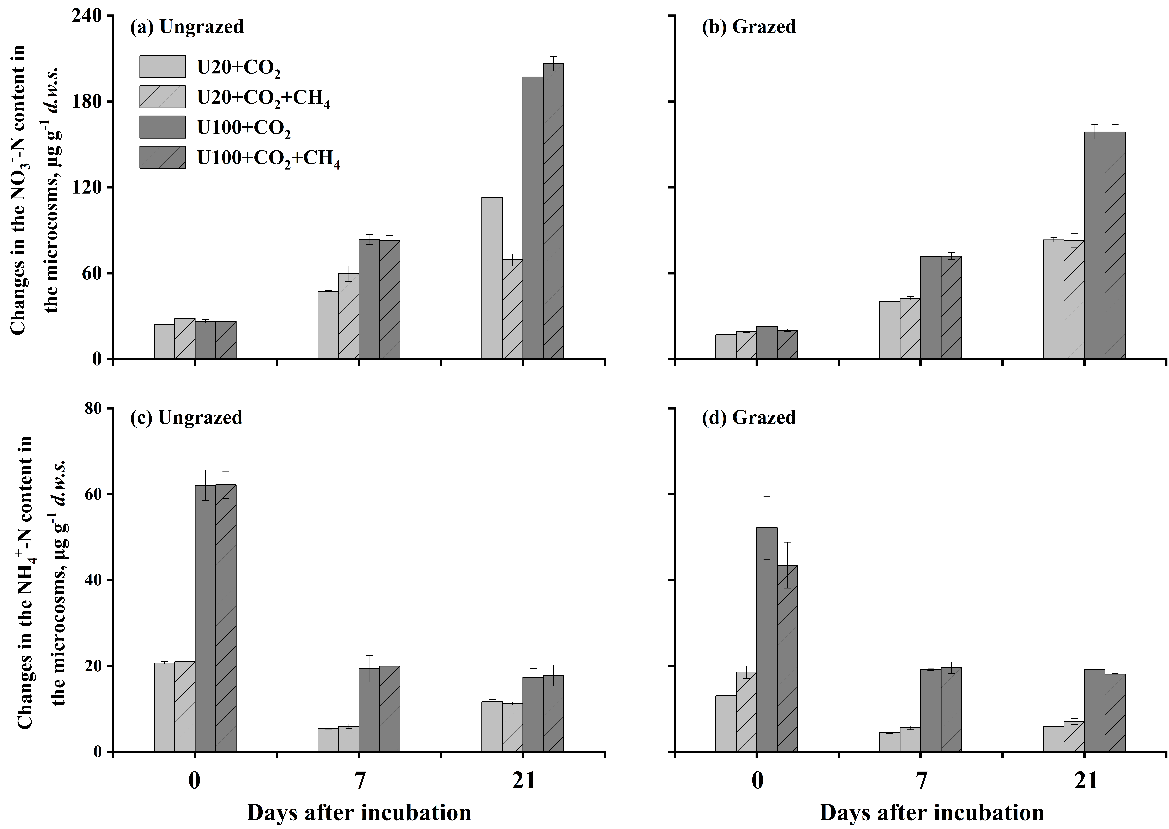


**Fig. S2** Changes in the soil NO_3_^-^-N (a, b) and NH_4_^+^-N (c, d) concentrations over a 21-day incubation period in the SIP microcosms. The error bars represent the standard errors of the mean of triplicate microcosms, while for the soil microcosms of Urea+CO_2_+CH_4_ treatments 6 replicates were used including both ^12^C-control and ^13^C-labeled treatments.


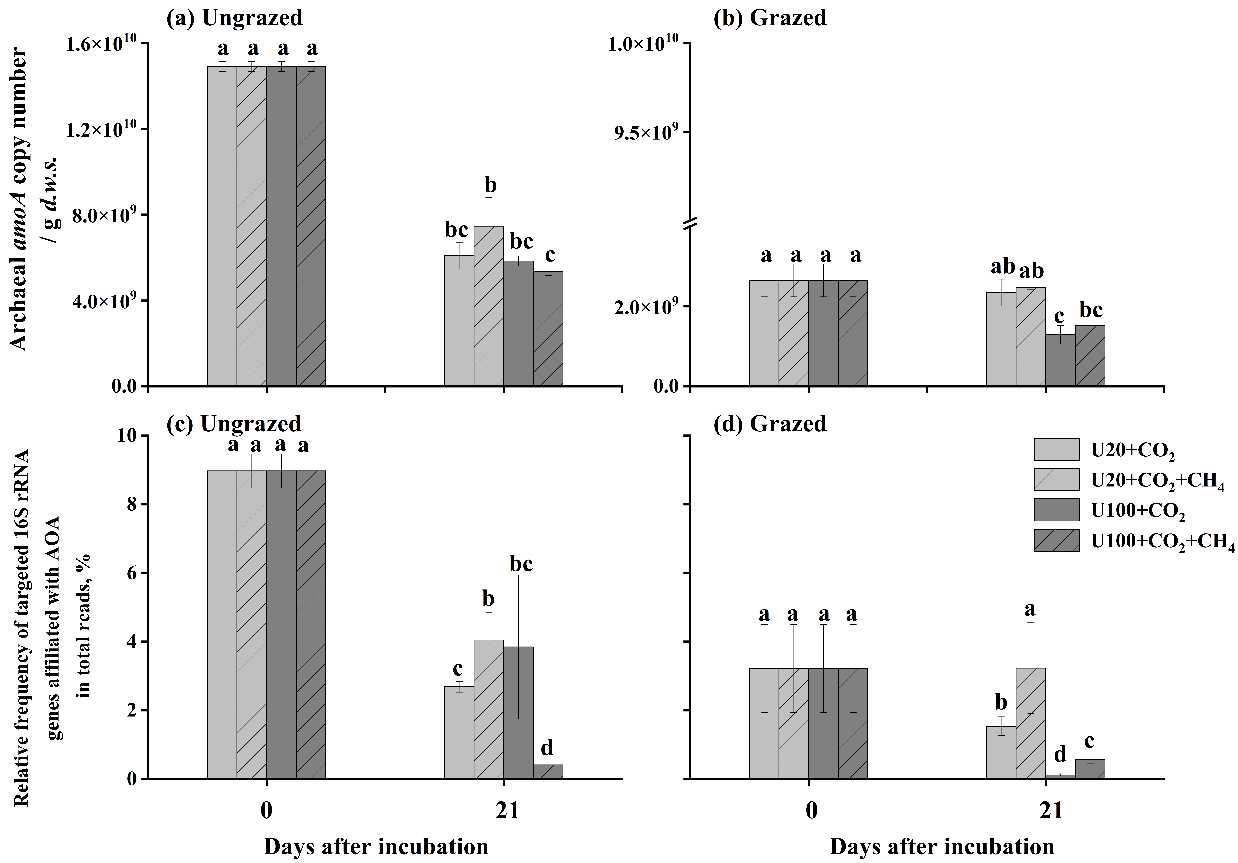


**Fig. S3** Changes of quantitative distribution of AOA *amoA* genes in soil microcosms over an incubation period of 21 days in ungrazed (a, c) and grazed (b, d) soils. The *amoA* gene copy numbers in total DNA from microcosms after incubation for 21 days in ungrazed (a) and grazed (b) soils; Relative frequencies of targeted 16S rRNA genes affiliated with AOA in total reads (%) in ungrazed (c) and grazed (d) soils. The error bars represent standard errors of 6 replicates including both ^12^C-control and ^13^C-labeled treatments.


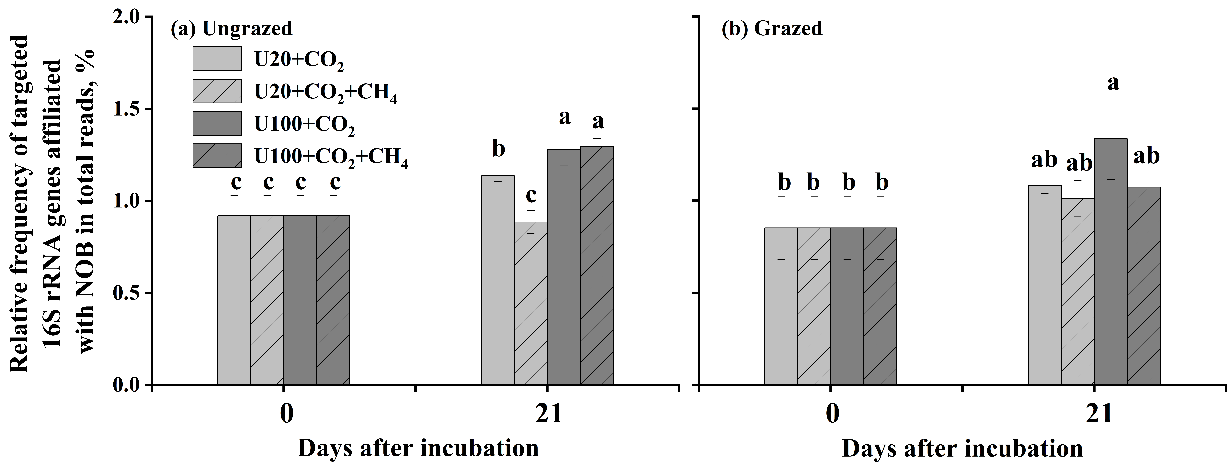


**Fig. S4** The relative abundances of NOB in ungrazed (a) and grazed (b) soils determined by high-throughput fingerprinting of 16S rRNA genes. The error bars represent the standard errors of the mean of triplicate microcosms, while for the soil microcosms of Urea+CO_2_+CH_4_ treatments 6 replicates were used including both ^12^C-control and ^13^C-labeled treatments.


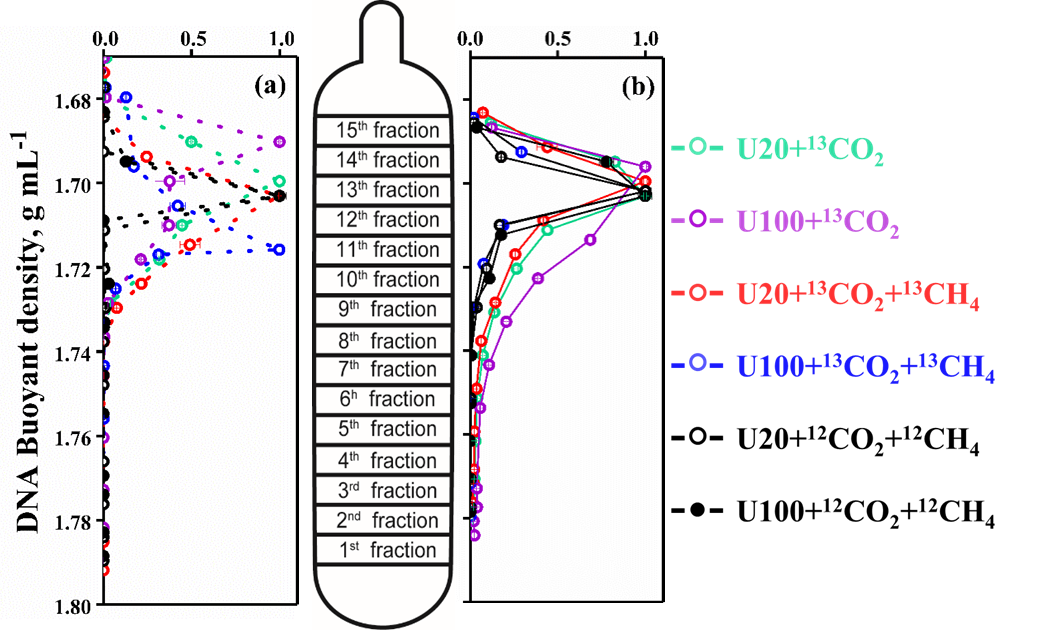


**Fig. S5** Quantitative distribution of archaeal *amoA* genes across the entire buoyant density gradient of the fractionated DNA from the ungrazed (a) and grazed (b) soils at weekly urea addition of 0 (U0), 20 (U20) and 100 μg N g^-1^ (U100), in presence or absence of methane addition (1% CH_4_) after incubation for 21 days. The normalized data are shown as the ratio of the gene copy number in each fraction to the maximum quantities in each treatment. The dotted and plain lines represent samples in ungrazed and grazed soils, respectively.


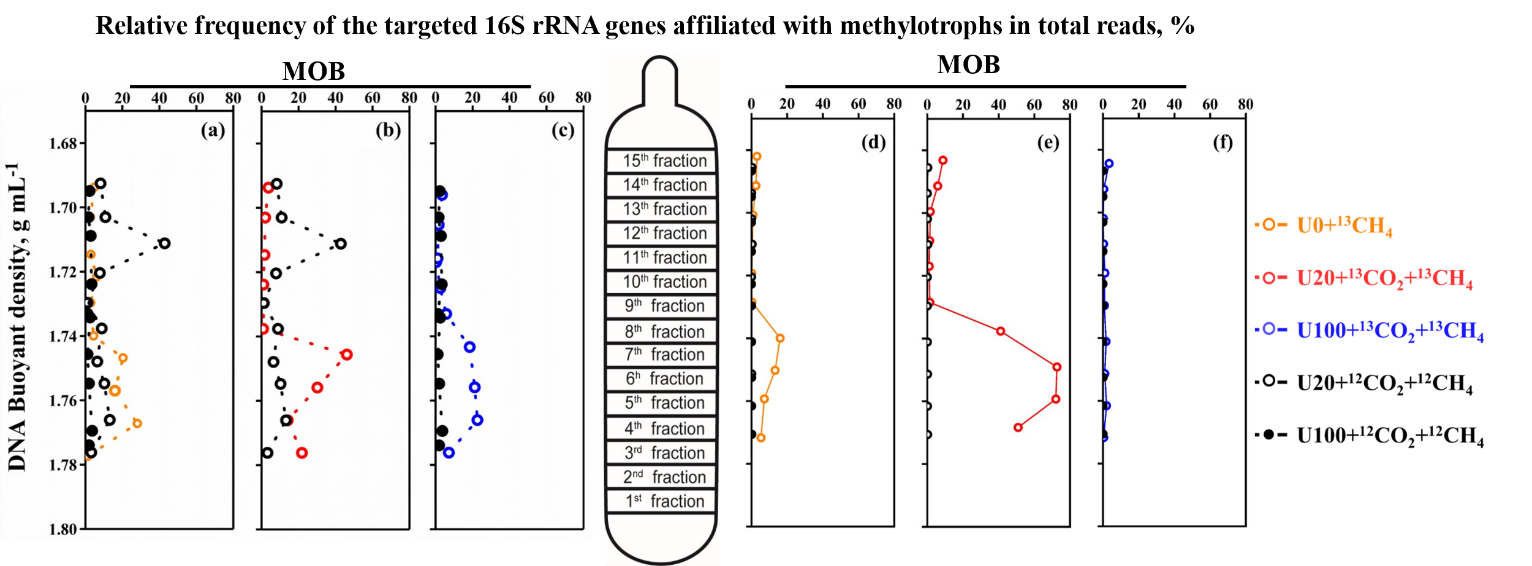


**Fig. S6** The relative frequency of the targeted 16S rRNA genes affiliated with methylotrophs in total reads. The ratios were calculated by targeted 16S rRNA gene reads affiliated with MOB to the total microbial 16S rRNA gene reads across the entire buoyant density gradient of the fractionated DNA from soil microcosms after incubation for 21 days in ungrazed (a-c) and grazed (d-f) soils.


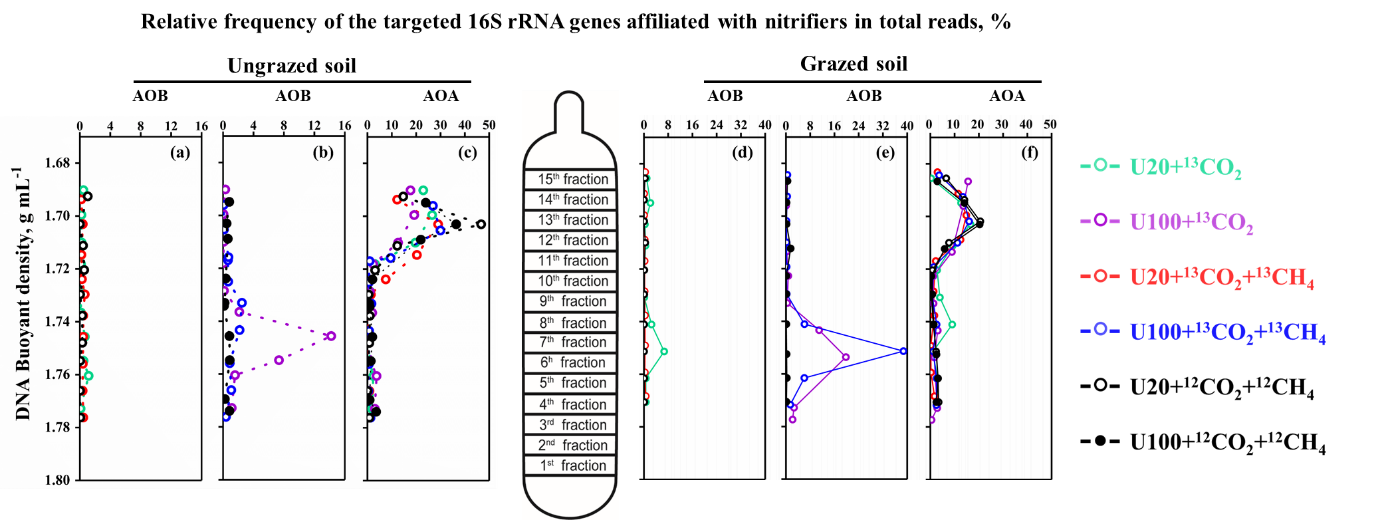


**Fig. S7** The relative frequency of the targeted 16S rRNA genes affiliated with nitrifiers in total reads. The ratios were calculated by targeted 16S rRNA gene reads affiliated with nitrifiers to the total microbial 16S rRNA gene reads across the entire buoyant density gradient of the fractionated DNA from soil microcosms after incubation for 21 days in ungrazed (a-c) and grazed (d-f) soils.


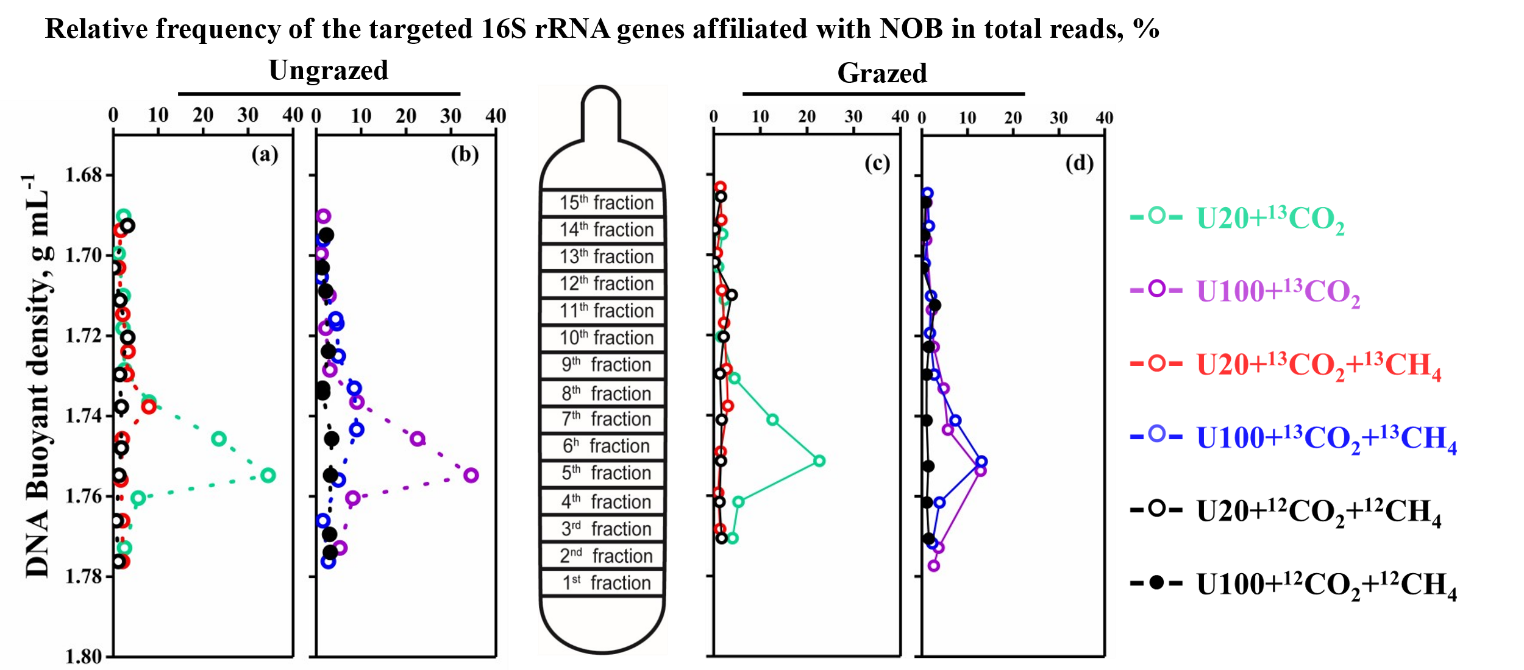


**Fig. S8** The relative frequency of the targeted 16S rRNA genes affiliated with NOB in total reads. The ratios were calculated by targeted 16S rRNA gene reads affiliated with NOB to the total microbial 16S rRNA gene reads across the entire buoyant density gradient of the fractionated DNA from soil microcosms after incubation for 21 days in ungrazed (a, b) and grazed (c, d) soils.


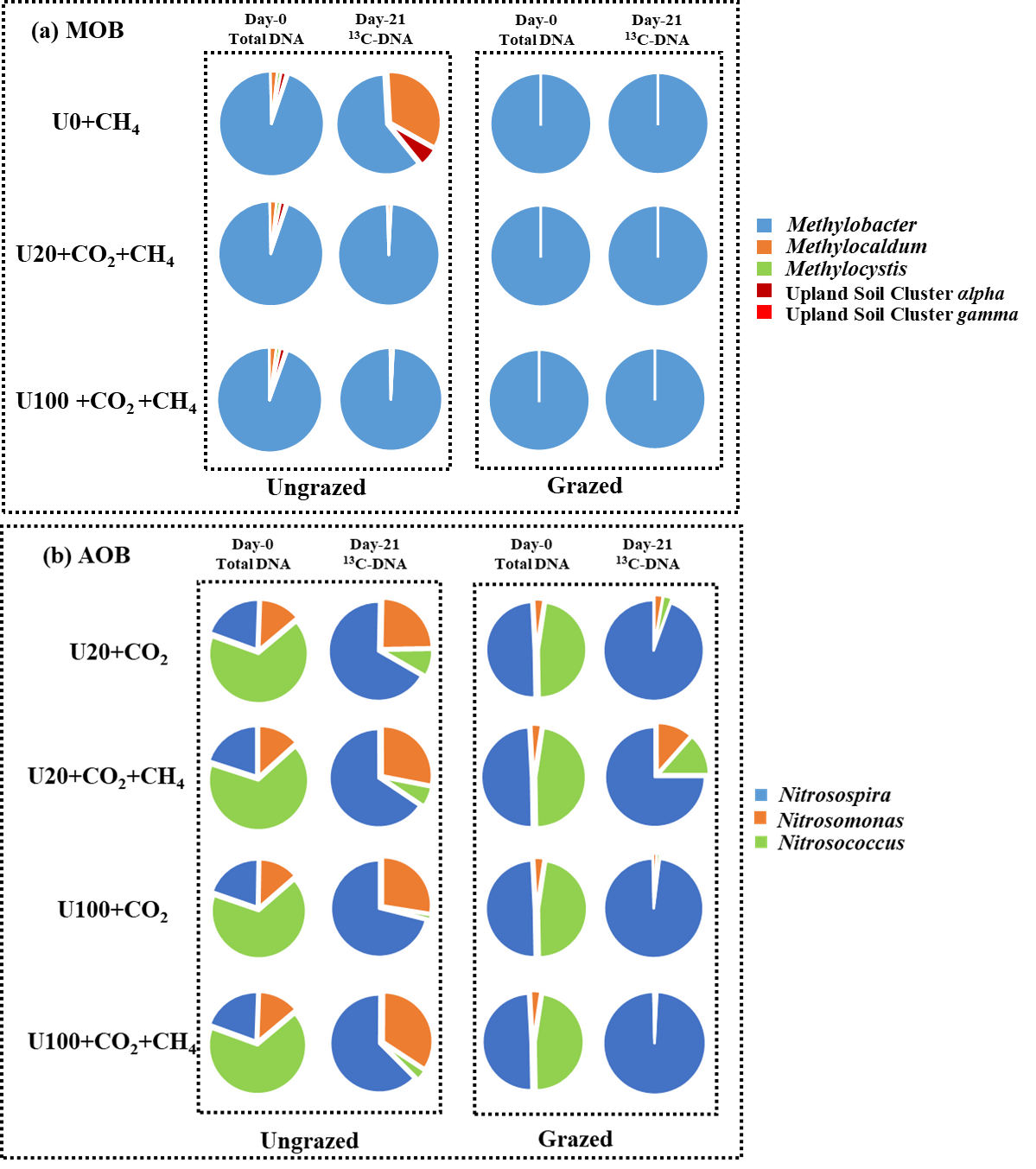


**Fig. S9** Proportional changes of methanotroph (a) and AOB (b) phylotypes in SIP microcosms after an incubation period of 21 days in Ungrazed and Grazed soils. The changes in active MOB and AOB composition were analyzed by target 16S rRNA genes in the active DNA from labeled microcosms at day 21.

**Supplementary References**

1. Francis CA, Roberts KJ, Beman JM, Santoro AE, Oakley BB. Ubiquity and diversity of ammonia-oxidizing archaea in water columns and sediments of the ocean. P Natl Acad Sci. 2005; 102: 14683-14688.
2. Rotthauwe JH, Witzel KP, Liesack W. The ammonia monooxygenase structural gene amoA as a functional marker: molecular fine-scale analysis of natural ammonia-oxidizing populations. Appl Environ Microbiol. 1997; 63: 4704-4712.
3. Kolb S, Knief C, Stubner S, Conrad R. Quantitative detection of methanotrophs in soil by novel pmoA-targeted real-time PCR assays. Appl Environ Microbiol. 2003; 69: 2423-2429.

4. Stubner S. Enumeration of 16S rDNA of Desulfotomaculum lineage 1 in rice field soil by real-time PCR with SybrGreen™ detection. J Microbial Meth. 2002; 50: 155-164.
